# Distinct intraspecies virulence mechanisms regulated by a conserved transcription factor

**DOI:** 10.1073/pnas.1903461116

**Published:** 2019-09-09

**Authors:** James P. R. Connolly, Nicky O’Boyle, Natasha C. A. Turner, Douglas F. Browning, Andrew J. Roe

**Affiliations:** ^a^Institute of Infection, Immunity and Inflammation, University of Glasgow, G12 8TA Glasgow, United Kingdom;; ^b^Institute for Cell and Molecular Biosciences, Newcastle University, NE2 4HH Newcastle-upon-Tyne, United Kingdom;; ^c^Institute of Microbiology and Infection, School of Biosciences, University of Birmingham, Edgbaston, B15 2TT Birmingham, United Kingdom

**Keywords:** regulation, gene expression, type 3 secretion, type 1 fimbriae, niche

## Abstract

Tailoring transcriptional regulation to coordinate the expression of virulence factors in tandem with the core genome is a hallmark of bacterial pathogen evolution. Bacteria encode hundreds of transcription factors forming the base-level control of gene regulation. Moreover, highly homologous regulators are assumed to control conserved genes between members within a species that harbor the same genetic targets. We have explored this concept in 2 *Escherichia coli* pathotypes that employ distinct virulence mechanisms that facilitate specification of a different niche within the host. Strikingly, we found that the transcription factor YhaJ actively regulated unique gene sets between intestinal enterohemorrhagic *E. coli* (EHEC) and extraintestinal uropathogenic *E. coli* (UPEC), despite being very highly conserved. In EHEC, YhaJ directly activates expression of type 3 secretion system components and effectors. Alternatively, YhaJ enhances UPEC virulence regulation by binding directly to the phase-variable type 1 fimbria promoter, driving its expression. Additionally, YhaJ was found to override the universal GAD acid tolerance system but exclusively in EHEC, thereby indirectly enhancing type 3 secretion pleiotropically. These results have revealed that within a species, conserved regulators are actively repurposed in a “personalized” manner to benefit particular lifestyles and drive virulence via multiple distinct mechanisms.

## Significance

Bacterial pathogens emerge by adapting mechanisms of virulence, differentiating them from their nonpathogenic progenitor. Virulence factors are often encoded on accessory genomic elements not part of the core genome and therefore must be integrated into the regulatory architecture of the cell. Here, we show that a highly conserved transcription factor in *Escherichia coli* has been relieved of a common purpose and adapted to regulate virulence pleiotropically in 2 distinct genetic backgrounds. This leads to enhanced virulence of both intestinal enterohemorrhagic *E. coli* and extraintestinal uropathogenic *E. coli* by exclusive mechanisms. These findings challenge the assumption that conserved transcription factors regulate common pathways maintained within a species and suggest that transcriptional repurposing creates new primary roles on an individual basis.

The regulation of gene expression is at the very heart of how a cell functions (1–3). Bacteria encode large numbers of diverse transcription factors that coordinate a plethora of regulatory roles. Indeed, it has been noted that *Escherichia coli* devotes ∼6% of its entire genome to regulatory genes, which in turn determine what genes are expressed and therefore what functions are performed (4). Many regulators are conserved between distinct members within a species, which can imply roles in the regulation of core-encoded genes. However, since the dawn of high-throughput genome sequencing, it has become apparent that the gene content of individual isolates can vary dramatically due to both genome minimization and the acquisition of horizontally acquired DNA (5). Thus, this creates a need to tailor the regulation of these genes at an individual level in order to appropriately coordinate gene expression (4).

Horizontally acquired DNA often encodes virulence factors that can transform harmless bacteria into pathogens capable of causing disease. *E. coli* has evolved a number of distinct pathotypes in this way (6). Enterohemorrhagic *E. coli* (EHEC) is a zoonotic pathogen capable of causing severe diarrheal illness in humans. This pathogenesis is facilitated by colonization of the colon using a type 3 secretion system (T3SS) encoded on a pathogenicity island known as the locus of enterocyte effacement (LEE) (7). This cellular attachment mechanism is independent of any specific tissue-receptor tropism and is instead governed by numerous transcriptional regulators in the cell, which converge on the LEE to control its expression in response to niche-specific signals such as nutrients, pH, and quorum-sensing molecules (8–10). Furthermore, this T3SS delivers non–LEE-encoded effector (NLE) proteins into host cells, which are encoded on cryptic prophages scattered throughout the genome, which must also be integrated into the global regulatory circuit of the cell (11).

In contrast, uropathogenic *E. coli* (UPEC) are capable of colonizing extraintestinal sites such as the urinary tract and kidneys (12). UPEC isolates, despite carrying a large number of genomic islands encoding virulence factors, heavily rely on type 1 fimbriae (T1F) to specifically bind mannosylated glycans found exclusively on the surface of the bladder epithelium, thus facilitating the first step in urovirulence (13, 14). Much like the T3SS, T1F are subject to regulation in response to environmental signals but the genetic basis of this is distinct (15). T1F are phase-variable, meaning their expression can be exclusively switched ON or OFF in individual cells by way of an invertible promoter upstream of the operon encoding the T1F apparatus, known as the Fim switch (16). Similar to the LEE, several regulators converge on this phase-variable region in order to fine-tune its expression appropriately. Importantly, T1F are widespread, encoded by nonpathogenic *E. coli* and even EHEC (17). However, EHEC isolates have a limited repertoire of functionally expressed fimbriae and T1F expression specifically is permanently silent in the EHEC O157:H7 clade specifically due to a 16-bp deletion in the Fim switch that locks the promoter in the OFF orientation (18, 19).

Conserved regulators are often assumed to have similar roles in a species where individual strains harbor the same set of target genes, while the regulation of genes specific to each individual can be considered an adapted role (4). We previously discovered that the highly conserved LysR-type transcriptional regulator (LTTR) YhaJ was adapted to directly activate T3SS expression in EHEC (20). LTTRs are the most diverse family of transcriptional regulators in the bacterial kingdom and we therefore hypothesized that it may play a role in controlling virulence of other *E. coli* pathotypes (21). Here, we have found that YhaJ binds distinct sites in the chromosomes of pathogenic EHEC and UPEC in vivo, including horizontally acquired regions. Furthermore, YhaJ regulates the expression of unique gene sets in these pathotypes, including not only strain-specific virulence factors but also core-encoded genes, thus challenging the assumption that conserved transcription factors share core functions within a species. In EHEC, YhaJ fine-tunes transcription of the T3SS and NLE virulence genes, whereas in UPEC, YhaJ stimulates positive phase variation and expression of T1F, thus representing 2 distinct mechanisms of virulence control for a single conserved regulator. Furthermore, YhaJ overrides the regulation of GAD acid tolerance in EHEC exclusively, pleiotropically regulating the T3SS via direct and indirect routes. This study demonstrates that intraspecies pathotypes have evolved to recycle conserved regulators in a “personalized” manner, creating tailor-made circuits of virulence regulation.

## Results

### Unique Gene Sets Regulated by a Universal Transcription Factor in Distinct *E. coli* Pathotypes.

**Fig. 1.**
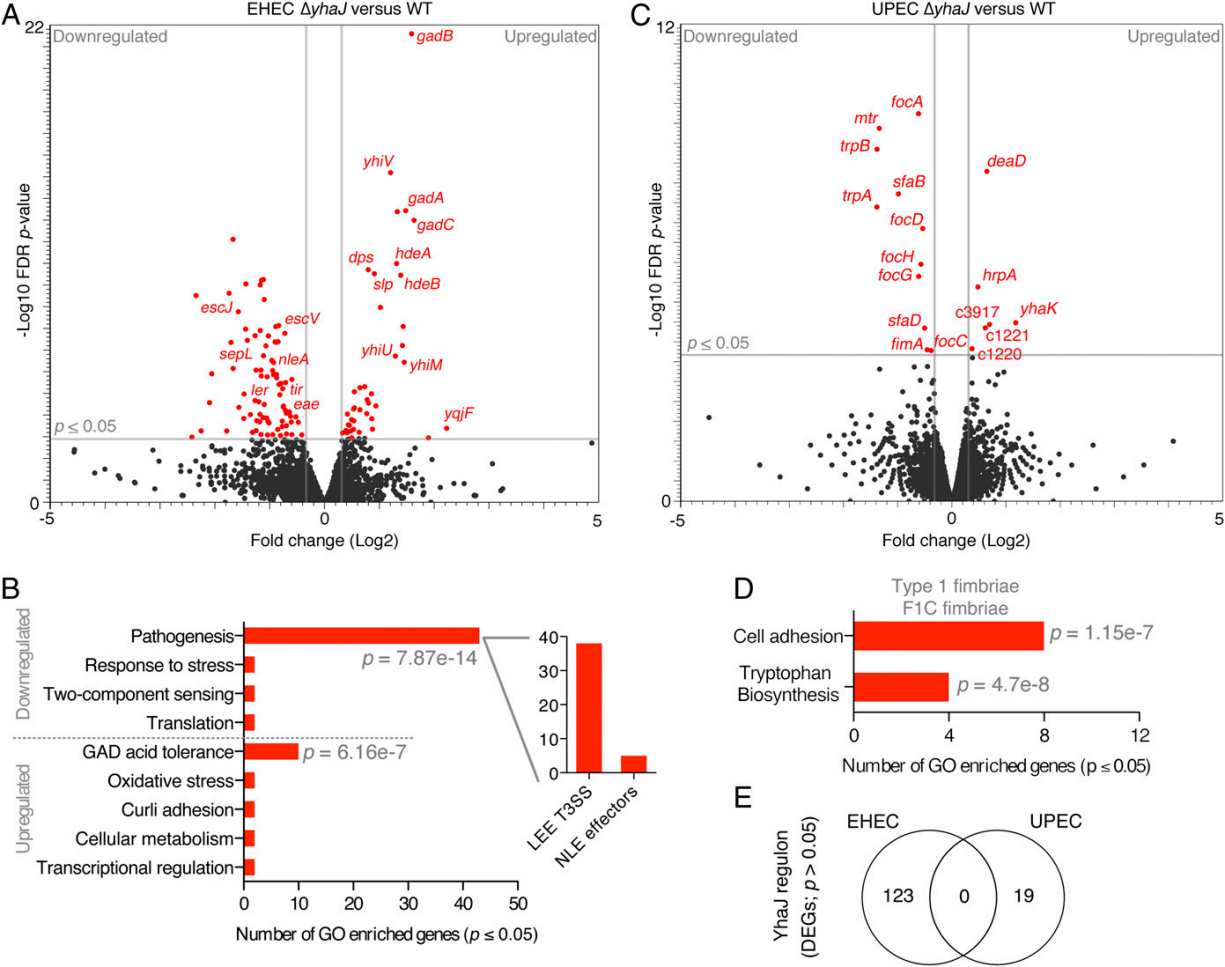
YhaJ regulon is unique in 2 distinct *E. coli* pathotypes. (*A*) Volcano plot of RNA-seq transcriptome data displaying the pattern of gene expression values for Δ*yhaJ* relative to WT EHEC cultured in MEM-Hepes (data analyzed from ref. 20). Significantly differentially expressed genes (FDR-corrected *P* ≤ 0.05) are highlighted in red, with the gray lines representing the boundary for identification of up- or down-regulated genes. Selected genes related to the T3SS, acid tolerance, and known YhaJ targets are indicated. (*B*) Gene ontology analysis of EHEC DEGs identifying significantly enriched functional categories (*P* ≤ 0.05). The major groups are highlighted, and the *Inset* graph depicts the number of genes related to the LEE island or non–LEE-encoded effectors. (*C*) Volcano plot of RNA-seq transcriptome data displaying the pattern of gene expression values for Δ*yhaJ* versus WT UPEC cultured identically as EHEC. (*D*) Gene ontology analysis of UPEC DEGs with the class of adhesion genes highlighted above. (*E*) Venn diagram comparing the YhaJ regulon of EHEC and UPEC illustrating the lack of any commonality in significant DEGs. All transcriptome experiments were performed in biological triplicate.

A hallmark of virulence regulation in EHEC is the recruitment of numerous transcription factors to fine-tune the expression of virulence in response to host signals, tailoring its circuits of regulation (9). These transcription factors are often core-encoded but can be adapted to control the expression of horizontally acquired virulence genes. Previously, comparative RNA-seq analysis of EHEC wild-type (WT) and Δ*yhaJ* cultured in MEM-Hepes minimal media identified significant (false discovery rate [FDR] *P* ≤ 0.05) differential expression of 123 genes, characterized largely by down-regulation of the entire LEE island (encoding a T3SS) as well as the non–LEE-encoded effector *nleA* (*P* = 1.55 × 10^−4^), which are both essential for virulence (Fig. 1*A* and *SI Appendix*, Table S1) (20, 22, 23). Gene ontology (GO) analysis of the remaining differentially expressed genes (DEGs) revealed that YhaJ regulates additional subsets of genes in EHEC, suggesting a global regulatory influence (Fig. 1*B*). Most significantly, the down-regulation of virulence coincided with the up-regulation of several genes of the GAD acid fitness network (*P* = 6.16 × 10^−7^) and differential expression of the known YhaJ target gene *yqjF* (24).

In order to determine if virulence regulatory adaptation of this highly conserved transcription factor was a unique feature of the EHEC pathotype, we compared the transcriptomes of the Δ*yhaJ* mutant in EHEC and UPEC (referred to as Δ*yhaJ*^*EHEC*^ and Δ*yhaJ*^*UPEC*^ herein) relative to their respective WT parents, cultured under identical conditions. Similar to Δ*yhaJ*^*EHEC*^, Δ*yhaJ*^*UPEC*^ was not defective for growth but displayed a much more restricted regulon than that of EHEC, with only 19 significant DEGs identified compared with the WT (Fig. 1*C* and *SI Appendix*, Fig. S1 and Table S2). The functional categories of these genes broke down into only 2 significantly enriched GO categories, type 1/F1C fimbrial adhesion (*P* = 1.15 × 10^−7^) and tryptophan biosynthesis (*P* = 4.7 × 10^−8^) (Fig. 1*D*). This was highly intriguing for 2 reasons. First, down-regulation of the *fimA* gene (*P* = 0.04) specifically belonging to the T1F gene cluster suggested that YhaJ regulates this key virulence determinant of the UPEC pathotype, critical for bladder colonization (25). Second, there was the striking observation that there were no overlapping DEGs common to both Δ*yhaJ*^*EHEC*^ and Δ*yhaJ*^*UPEC*^ regulons, under identical conditions (Fig. 1*E*). This shows that despite being highly conserved, YhaJ has been recycled to regulate the expression of distinct gene sets, including key virulence factors, in both EHEC and UPEC genetic backgrounds.

### YhaJ Targets Unique Chromosomal Binding Sites in EHEC and UPEC Pathotypes.

**Fig. 2.**
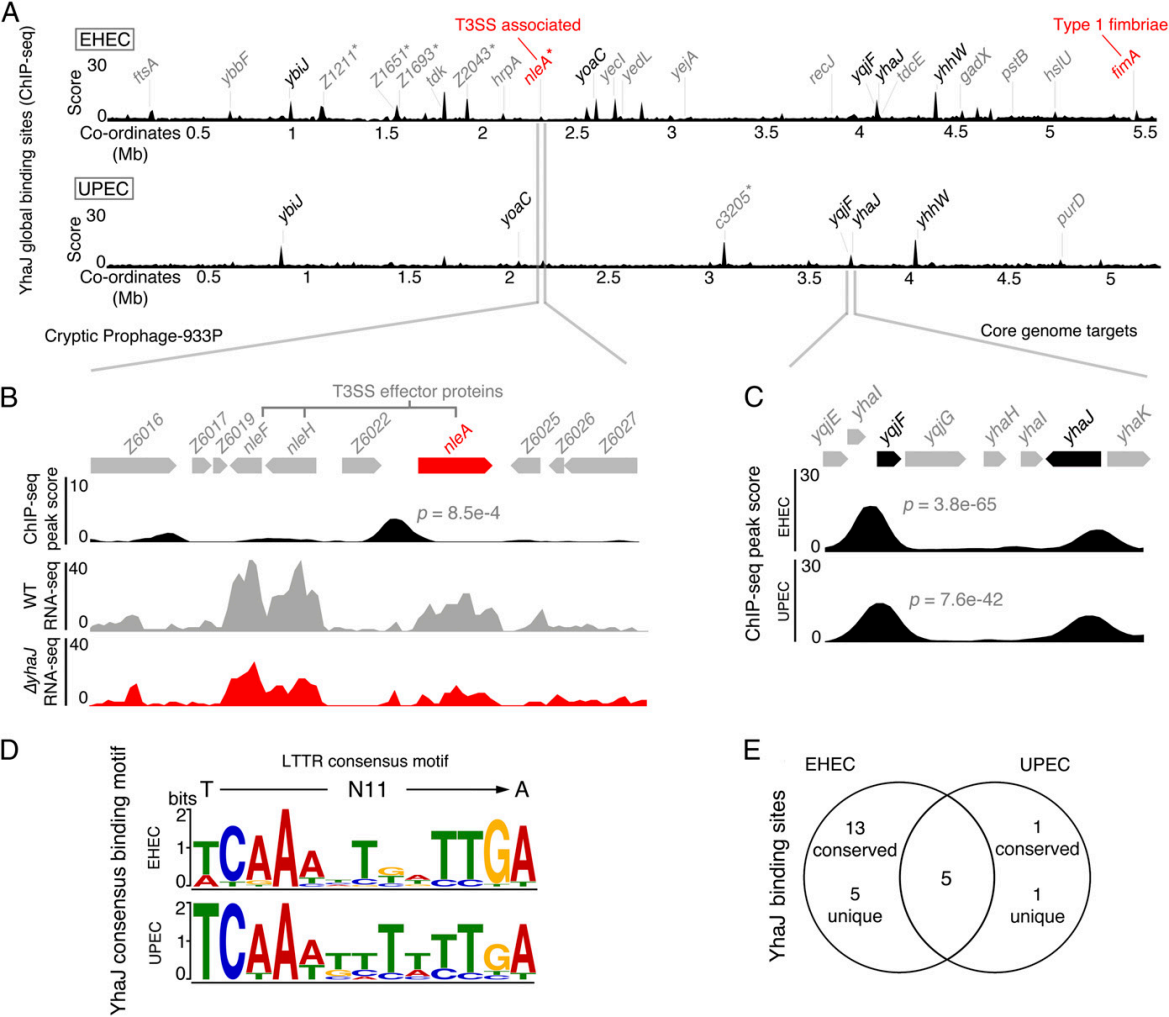
YhaJ binds to unique core and horizontal chromosomal locations in distinct *E. coli* pathotypes. (*A*) Global chromosome binding dynamics of YhaJ in EHEC and UPEC identified by ChIP-seq. Significant peaks (*P* ≤ 0.01; 2 biological replicates) related to known targets are labeled in black, novel targets are in gray, and pathotype-specific targets are marked with an asterisk. The virulence-associated targets *nleA* (T3SS-associated) and *fimA* (type 1 fimbriae) are highlighted in red. (*B*) Expanded view of the horizontally acquired CP-933P locus in EHEC. The prophage region encodes multiple non–LEE-encoded effectors as indicated. The ChIP-seq track illustrates the identified YhaJ binding site upstream of *nleA* and RNA-seq data illustrate the downshift in transcription from *nleA* for Δ*yhaJ* (red) versus WT EHEC (gray). (*C*) Expanded view of ChIP-seq peaks identified for 2 common core genome targets, *yhaJ* itself and *yqjF.* The *P* values for *yqjF* peaks are indicated. (*D*) The computed consensus binding motif for YhaJ targets in EHEC and UPEC based on ChIP-seq data. The motifs match the generalized consensus sequence for LTTRs (T-N11-A) as illustrated above. (*E*) Comparison of YhaJ binding sites in EHEC and UPEC. ChIP-seq experiments were performed in biological duplicate.

In order to take a deeper look at the molecular mechanisms governing these pathotype-specific YhaJ regulons, we investigated the functionality of this transcription factor in vivo using chromatin immunoprecipitation sequencing (ChIP-seq) analysis. YhaJ shares >99% sequence identity (298/299 residues) in both EHEC and UPEC, with complete conservation in the DNA- and substrate-binding domains in both strains (*SI Appendix*, Fig. S2). We chose to investigate each natively expressed protein by engineering strains encoding 3× FLAG-tagged YhaJ in both EHEC and UPEC genetic backgrounds. We determined the global binding profile of YhaJ by enriching for chromosomal DNA immunoprecipitated with natively expressed FLAG-tagged YhaJ, identifying specific motif regions associated with these targets (*SI Appendix*, Fig. S3). This approach uncovered a unique YhaJ target map for each strain (Fig. 2*A*). In EHEC, YhaJ bound significantly to 23 chromosomal locations (*SI Appendix*, Table S3). These targets included known regulated genes (*ybiJ*, *yoaC*, *yqjF*, *yhaK*, and *yhhW*) but also targets found both on the core *E. coli* genome (13 targets including the GAD regulator gene *gadX*) as well as 5 EHEC-specific sequences (*SI Appendix*, Fig. S4) (24, 26). Strikingly, these EHEC targets were located within cryptic prophage regions and upstream of genes encoding the pathotype-specific virulence factor *nleA* (*P* = 8.5 × 10^−4^) and 2 identical autotransporters, *Z1211* and *Z1651*, identified previously as Ag43 homologs Cah1 and Cah2 (Fig. 2*B*) (27). As discussed above, *nleA* is down-regulated in the Δ*yhaJ*^*EHEC*^ background, with a binding site situated farther away from the gene than previously predicted (20). Indeed, binding sites of all YhaJ targets did not follow a strict positional bias relative to the nearest start codon; however, a majority of ChIP-seq peaks clustered to promoter regions upstream of the nearest gene (*SI Appendix*, Fig. S5). We validated the binding of YhaJ to the extended *nleA* promoter region in vitro by electrophoretic mobility shift assay (EMSA) analysis and confirmed the negative regulation of this gene using an *nleA*::GFP reporter (*P* = 0.0129) in the Δ*yhaJ*^*EHEC*^ background, which we successfully complemented in trans (*SI Appendix*, Fig. S6). These data correlate well with the RNA-seq analysis in identifying directly regulated targets such as several virulence factors, the GAD acid tolerance response, and the known target *yqjF* (24).

In stark contrast to EHEC, only 7 significant YhaJ binding sites were identified in UPEC under these conditions (*SI Appendix*, Table S4). Five of these binding sites were the same known YhaJ targets identified in EHEC, with similar signal enrichment (Fig. 2*C* and *SI Appendix*, Fig. S4). Two unique targets were identified, *purD* and c3206, the latter of which is a UPEC-specific hypothetical gene. Binding of YhaJ to pathotype-specific sequences was validated by EMSA analysis, demonstrating the possibility of further pathotype-specific regulatory mechanisms that are unclear under the conditions used in this study (*SI Appendix*, Fig. S7). It should be noted that all EMSAs were performed using EHEC YhaJ, confirming that the single-amino acid change in the coding sequence was not responsible for the differential binding profiles in vitro. Analysis of the peak sequences using Multiple EM for Motif Elicitation revealed almost identical predicted YhaJ motifs in both EHEC and UPEC, with high significance (*E* = 5.5 × 10^−15^) in comparison with the predicted YhaJ motif, and also matched the canonical LTTR motif structure of T-N11-A (Fig. 2*D*) (21). However, despite this similarity, only 5 of the total binding sites identified were present in both strains (Fig. 2*E*). Surprisingly, none of the UPEC ChIP-seq targets were found to exhibit differential transcript levels under these conditions (RNA-seq). This suggests that different pathotypes have adapted a suite of direct targets capable of being regulated by YhaJ, including horizontally acquired genes, which are not uniformly controlled under 1 common condition. Furthermore, investigation of *yhaJ* expression using a *yhaJ*::GFP promoter-fusion reporter revealed transcription levels of this regulator are >2-fold lower in UPEC than EHEC, despite their promoter sequences being identical (*SI Appendix*, Fig. S8*A*). Immunoblot analysis probing for the FLAG epitope in whole-cell lysates from both strains confirmed that expression of YhaJ in EHEC is substantially higher than in UPEC (*SI Appendix*, Fig. S8*B*). However, ChIP-PCR analysis of 2 common YhaJ targets (*yhaK* and *yqjF*) revealed comparable signal-to-noise enrichment ratios of binding to these gene promoter regions in both strains (*SI Appendix*, Fig. S8*C*). These results suggest that YhaJ has the potential to target common genes in both pathotypes but that its own regulation is unique in each genetic background. Nonetheless, it is apparent that a key role of YhaJ is to tailor the transcriptional program of individual pathotypes, which leads to differential expression of key virulence genes.

### YhaJ Overrides the Regulation of EHEC Acid Tolerance to Control the T3SS via Direct and Indirect Routes.

**Fig. 3.**
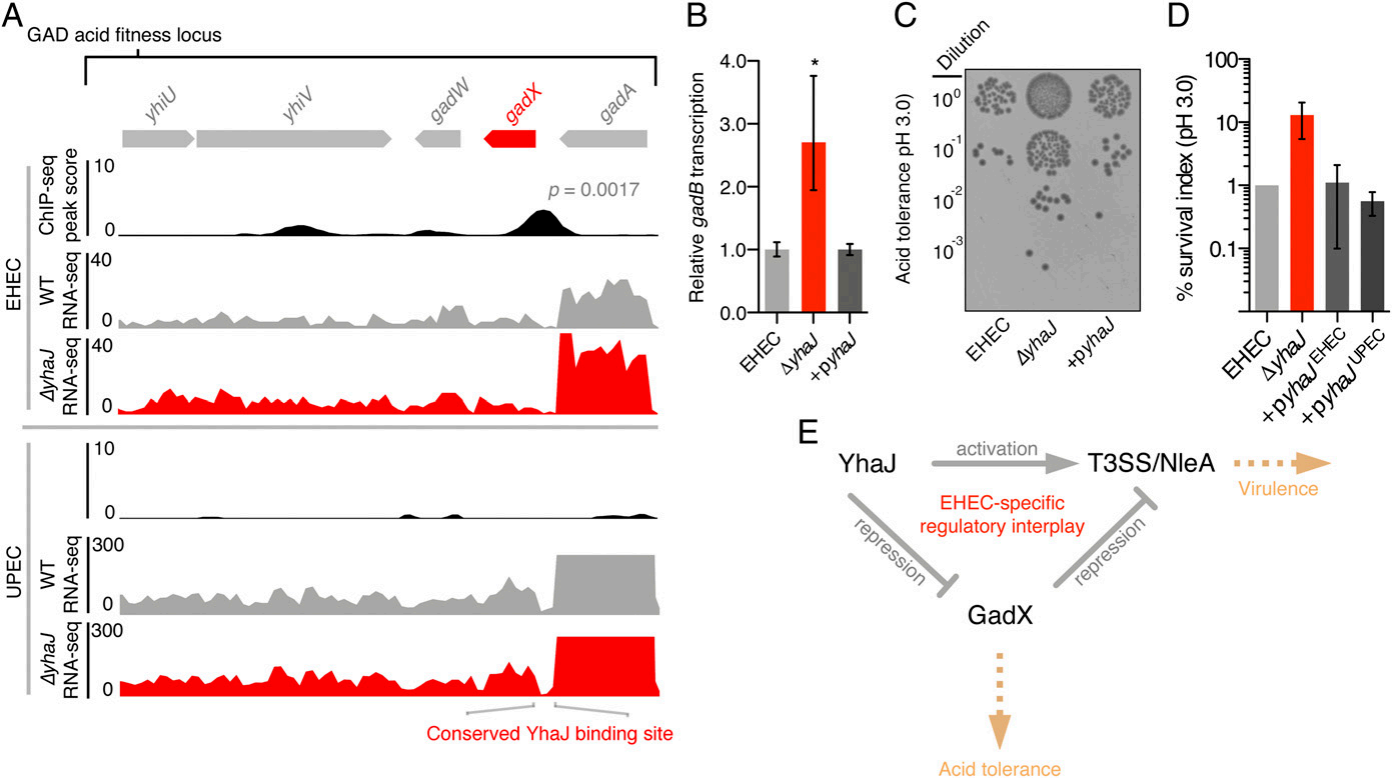
YhaJ overrides the regulation of EHEC acid tolerance to control virulence via direct and indirect routes. (*A*) Expanded view of the EHEC YhaJ binding site upstream of the GAD acid tolerance regulator gene *gadX*. (*A*, *Upper*) RNA-seq tracks show the upshift in GAD gene transcription for Δ*yhaJ* (red) versus WT EHEC (gray). (*A*, *Lower*) Tracks reveal the lack of this regulation in a UPEC genetic background. The conserved sequence of the YhaJ-binding motif in both strains is illustrated (*Bottom*). (*B*) Repression of acid tolerance by YhaJ confirmed by qRT-PCR analysis of the GadX-regulated gene *gadB* in WT EHEC, Δ*yhaJ*, and +p*yhaJ* backgrounds. *P ≤ 0.05 derived from 3 biological replicates ±SD (Student’s *t* test). (*C*) Acid tolerance assay of WT EHEC, Δ*yhaJ*, and complemented (+p*yhaJ*) cells exposed to acidic conditions (pH 3.0). (*D*) Quantification of CFUs from *B* representing the mean survival relative to the WT (±SD of 4 biological replicates). The EHEC Δ*yhaJ* phenotype was complemented with both the EHEC and UPEC *yhaJ* alleles expressed in trans (+p*yhaJ*^EHEC^ and +p*yhaJ*^UPEC^, respectively). (*E*) The regulatory interplay between YhaJ and GadX in fine-tuning T3SS expression in EHEC. Pointed and blunt arrows represent activation and repression, respectively, whereas the yellow arrows indicate the associated phenotype of each pathway.

Natively expressed YhaJ was found to bind the *gadX* promoter region in EHEC but not UPEC. This was highly interesting, given the significant impact that the Δ*yhaJ* deletion had on GAD gene expression in EHEC exclusively (Fig. 3*A*). We therefore hypothesized that YhaJ indirectly controls acid tolerance via GadX in EHEC. First, we confirmed the binding of YhaJ specifically upstream of *gadX* by EMSA analysis (*SI Appendix*, Fig. S9). Next, we validated the repression of the GAD system in EHEC by qRT-PCR analysis. Δ*yhaJ*^*EHEC*^ displayed an approximately 2.5-fold increase in expression of the GadX-regulated gene *gadB* (*P* = 0.037), whereas complementation restored WT levels of *gadB* expression (Fig. 3*B*). To test this functionally, we exposed EHEC WT and Δ*yhaJ*^*EHEC*^ cells to media preconditioned to pH 3.0. This experiment revealed a marked increase in the number of Δ*yhaJ*^*EHEC*^ colony-forming units (CFUs) returned, a phenotype that was successfully complemented (Fig. 3*C*). Quantification of CFU counts relative to WT survival in acidified media showed that deletion of *yhaJ* increased the percentage of survival by >∼10-fold, in line with the transcriptional shift in GAD expression observed by RNA-seq (Fig. 3*D*). In contrast, we observed no dependency on YhaJ in UPEC for regulating acid tolerance. The Δ*yhaJ*^*UPEC*^ strain displayed no differential regulation of *gadB* by qRT-PCR and no enhanced survival in response to acidified media was detected (*SI Appendix*, Fig. S10). Importantly, we confirmed that this phenotypic distinction in EHEC was not driven by the single-amino acid change in the YhaJ protein sequence by successfully complementing the Δ*yhaJ*^*EHEC*^ phenotype using the UPEC *yhaJ* allele expressed in trans (Fig. 3*D*). Similarly, expression of the EHEC *yhaJ* allele in the Δ*yhaJ*^*UPEC*^ strain had no additive phenotype (*SI Appendix*, Fig. S10). Intriguingly, despite the lack of ChIP-seq signal, YhaJ was capable of binding to the UPEC *gadX* promoter region in vitro by EMSA analysis in accordance with the predicted binding motif and promoter being identical between both pathotypes, suggesting that regulation of the EHEC cell circuit has been specifically tailored in vivo to control *gadX* directly by YhaJ, independent of any mutation in the coding or binding sequence (*SI Appendix*, Fig. S11). The data described here have revealed that YhaJ has been recruited to enhance T3SS expression in EHEC by 2 distinct mechanisms—direct activation of T3SS/prophage-encoded effector expression as well as direct repression of the GAD system, which in turn promotes T3SS expression indirectly (Fig. 3*E* and *SI Appendix*, Fig. S12).

### YhaJ Positively Regulates T1F Expression in Uropathogenic *E. coli*.

**Fig. 4.**
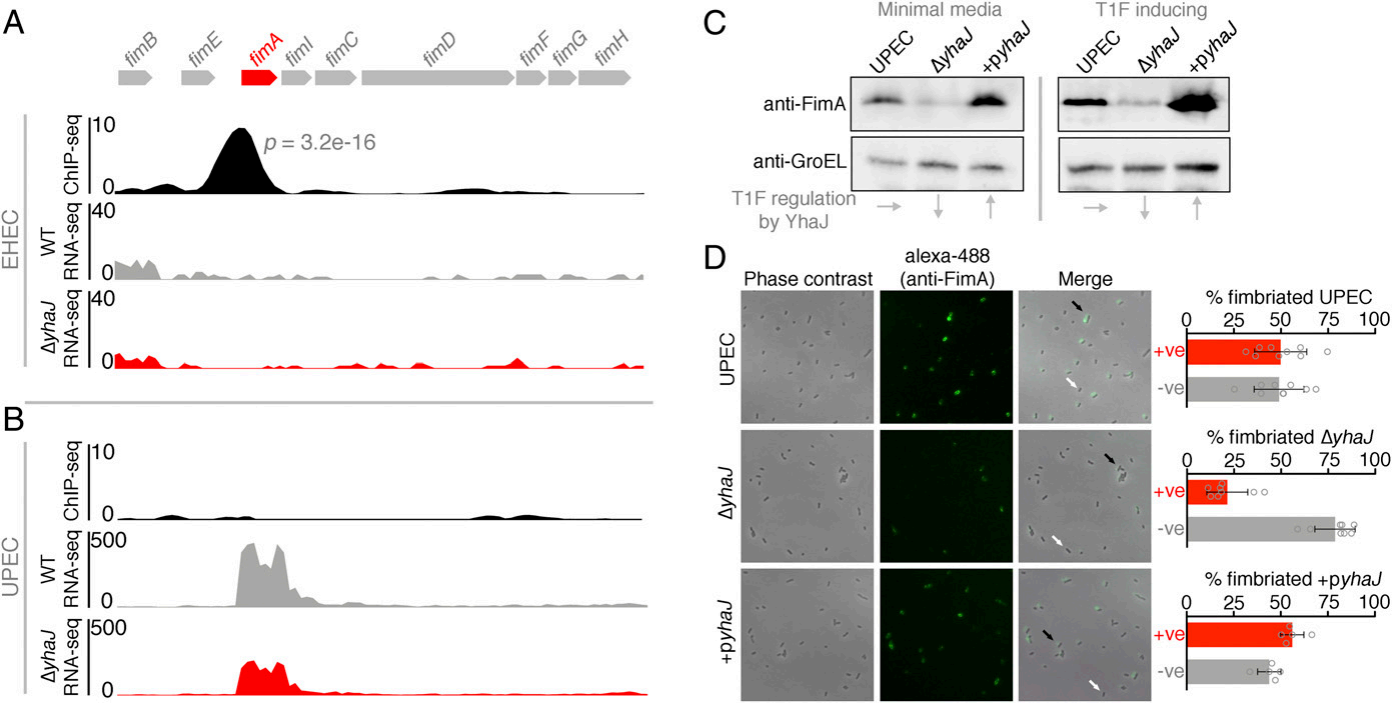
YhaJ enhances expression of type 1 fimbriae in UPEC. (*A*) Expanded view of the YhaJ binding site upstream of the silent T1F locus in EHEC identified by ChIP-seq. The associated RNA-seq tracks highlight the lack of transcription from this silent locus in both WT EHEC (gray) and Δ*yhaJ* (red). (*B*) ChIP-seq data from the active T1F locus in UPEC illustrating the lack of YhaJ enrichment but a downshift in *fimA* transcription for Δ*yhaJ* (red) versus WT UPEC (gray). (*C*) Immunoblot analysis of FimA expression levels from WT UPEC, Δ*yhaJ*, and the complemented mutant +p*yhaJ* grown in minimal media (ChIP/RNA-seq conditions) and T1F-inducing conditions (static LB at 37 °C). Levels of GroEL were used to assess equal loading, and the influence of *yhaJ* mutation/complementation on T1F expression is highlighted (*Bottom*). Multiple biological replicates of immunoblots were performed. (*D*) Phase-contrast microscopy of WT UPEC, Δ*yhaJ*, and +p*yhaJ* grown under T1F-inducing conditions overlaid with immunofluorescence images of the cells probed using anti-FimA and Alexa-488 antibodies. The level of fimbriation around single cells was assessed (black arrows, T1F-positive; white arrows, T1F-negative) from more than 5 random fields of view per replicate and expressed as percentage fimbriated cells within the population (*Right*). Error bars represent SD, and the experiments were performed on 3 independent occasions.

RNA-seq analysis of Δ*yhaJ*^*UPEC*^ revealed that this transcription factor is responsible for regulating T1F in UPEC (Fig. 1*B*). However, our attention was immediately drawn to our ChIP-seq analysis, which identified a strong YhaJ binding site (*P* = 3.2 × 10^−16^) upstream of the *fimA* gene in EHEC but not in UPEC, where enrichment was weak and almost completely absent in 1 replicate (Fig. 4 *A* and *B*). This seemed paradoxical, considering the fact that T1F are critical for UPEC virulence but are not expressed by EHEC. To confirm this unusual regulatory role, we performed immunoblot analysis probing for FimA levels from whole-cell lysates of UPEC cells grown in both minimal media (RNA/ChIP-seq conditions) and static lysogeny broth (LB) (T1F-inducing conditions). The results confirmed that the downshift in *fimA* transcription correlated with an ∼2.5-fold decrease in FimA protein levels for the Δ*yhaJ*^*UPEC*^ mutant (Fig. 4*C*). Importantly, complementation of this deletion restored and enhanced FimA expression relative to the WT. Furthermore, this phenotype was conserved under T1F-inducing conditions, affirming our proposal above that YhaJ tailors the virulence program of distinct pathotypes without conditional bias.

Expression of T1F in *E. coli* is phase-variable, driven by an invertible *fimS* promoter element upstream of *fimA* (16). This means that within any given population of cells, a certain proportion will express T1F (phase ON) whereas the remainder will not (phase OFF). To examine the regulation of T1F by YhaJ at the single-cell level, we used immunofluorescence microscopy probing specifically for FimA expression (Fig. 4*D*). The data showed that when grown in T1F-inducing conditions, ∼50% of the population was phase ON for T1F. However, in the Δ*yhaJ*^*UPEC*^ background there was a large downshift in T1F-expressing cells (∼20% phase ON), which correlated with the transcriptome and immunoblot analysis. Complementation of YhaJ restored the phenotype, resulting in an increase in T1F-expressing cells to ∼60% of the population. These data confirm that the regulation of T1F by YhaJ at the molecular level manifests in the production of T1F at the single-cell level.

The majority of *E. coli* isolates, pathogenic or not, possess T1F (17). To test if the regulation of T1F is a widely used mechanism in *E. coli*, we performed qRT-PCR analysis of *fimA* transcription in nonpathogenic *E. coli* K-12 (*SI Appendix*, Fig. S13*A*). Deletion of *yhaJ* in a K-12 background had no significant impact on *fimA* transcription, whereas in Δ*yhaJ*^*UPEC*^ the downshift was statistically significant (*P* = 0.0317). However, complementation of both Δ*yhaJ*^*UPEC*^ and Δ*yhaJ*^*K-12*^ resulted in enhanced expression of *fimA* (*P* ≤ 0.005). Immunoblot analysis of FimA expression in K-12 mimicked the transcriptional data, characterized by the ability of YhaJ to regulate T1F expression only when overexpressed in trans. Furthermore, we confirmed that growth conditions were not responsible for this phenomenon (*SI Appendix*, Fig. S13*B*). Collectively, these results have revealed that YhaJ is a functional regulator of T1F in distinct pathogenic and nonpathogenic *E. coli* isolates. However, pathogenic UPEC have evolved a dependency to specifically regulate T1F by YhaJ in order to achieve full expression of this virulence factor, further highlighting the potential for tailored regulatory reprogramming to have an impact on expression of key virulence genes.

### Orientation-Specific Binding of YhaJ to the *fimS* Element Promotes Promoter Inversion.

**Fig. 5.**
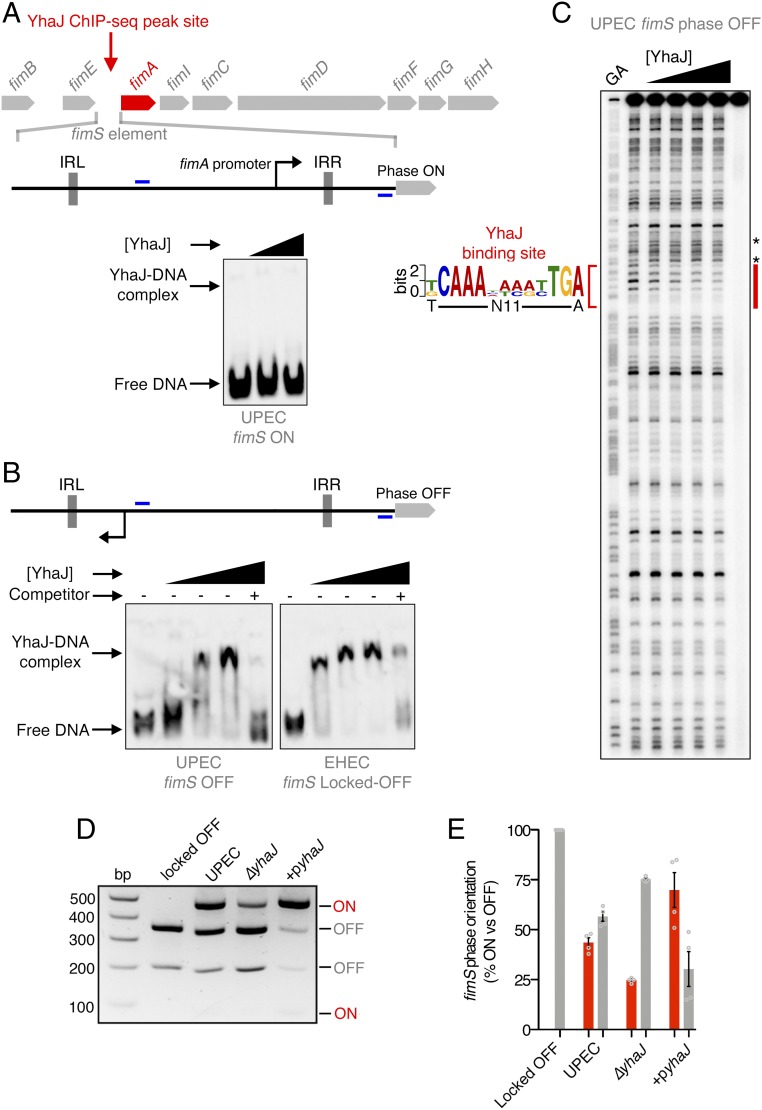
YhaJ promotes activation of the *fimS* element by binding exclusively in the OFF orientation. (*A*) Schematic depiction of the phase-variable *fimS* element, containing an invertible promoter, in the ON orientation. EMSA analysis showing that YhaJ does not bind UPEC *fimS* in the ON orientation. (*B*) Schematic depiction of *fimS* in the OFF orientation. EMSAs demonstrating YhaJ binding over increasing concentrations to both UPEC and EHEC exclusively in the OFF orientation (*Bottom*). Excess unlabeled probe was added as a competitor to demonstrate binding specificity. Positions of primers used to amplify EMSA probes are indicated in blue. (*C*) DNaseI footprinting analysis of YhaJ bound to *fimS*. The protected region is indicated in red with the corresponding YhaJ/LTTR sequence motif indicated. The asterisks denote canonical hypersensitive sites flanking the protected region. (*D*) T1F phase-switching assay used to demonstrate YhaJ-activated inversion of *fimS*. The *fimS* locus contains a single *Hinf*I restriction site, and DNA from cultures of WT UPEC, Δ*yhaJ*, and +p*yhaJ* was digested with *Hinf*I and analyzed on 2% agarose. The banding pattern indicates the abundance of the population expressing T1F ON (red) or OFF (gray). WT EHEC which harbors a locked-OFF *fimS* was used as a control. (*E*) Quantification of T1F phase-switching assays illustrates the relative percentage of the population expressing *fimS* ON or OFF. Data represent the mean of 4 biological replicates ±SD.

The paradoxical lack of YhaJ enrichment at the UPEC *fimS* region prompted questions about the mechanism of T1F regulation by this transcription factor. Given the strong YhaJ binding to the defective EHEC *fimS* element, we hypothesized that recruitment of YhaJ to *fimS* may occur exclusively when the promoter is orientated in the OFF phase. To test this, we performed EMSA analysis using primers that flanked the invertible element and the *fimA* intergenic region to amplify DNA probes of *fimS* in either the ON or OFF orientation exclusively. The analysis revealed that YhaJ was unable to bind UPEC *fimS* in the ON orientation (Fig. 5*A*). Conversely, EMSA analysis of both UPEC and EHEC *fimS* in the OFF orientation showed YhaJ binding specifically to this region, proving that the binding was indeed *fimS* orientation-specific (Fig. 5*B*). This experiment suggested that YhaJ specifically targets *fimS* exclusively in the phase OFF orientation, explaining why the ChIP-seq data (Fig. 2*A*) revealed strong signal to noise at this site in EHEC but not in UPEC, where the cell population is heterogeneous in the orientation of *fimS*.

The design of the EMSA probes included both phase-specific *fimS* DNA but also flanking sequence found in the *fimS* and *fimA* intergenic region. This suggested that there was possibly interaction between multiple sequence elements both within *fimS* and the intergenic region required to facilitate YhaJ binding. To determine the exact binding site identified by ChIP-seq, we used DNaseI footprint analysis of YhaJ bound to phase OFF *fimS* from UPEC (Fig. 5*C*). This analysis identified a single precise zone of protection that was immediately preceded by characteristic hypersensitive digestion residues, revealing a binding site within *fimS* that significantly matched the YhaJ consensus motif and was located close to the ChIP-seq peak center (*P* = 9.66 × 10^−7^). To confirm the specificity of this interaction, we designed 2 variants of the *fimS* OFF EMSA probes, mutating both the 5′ (C→T at position 2) and the 3′ (TGA→CCC at position 11 to 13) termini of the binding site, thus eliminating critical residues in the dyad symmetry of the sequence. EMSA analysis showed that mutation of either residue essentially eliminated the ability of YhaJ to bind the *fimS* element, thereby confirming the identification of YhaJ binding to this site exclusively in the phase OFF orientation (*SI Appendix*, Fig. S14).

Multiple transcription factors are employed in the regulation of T1F (15). However, the influence of regulators at the *fimS* site is often mediated by modulating expression of the Fim recombinases rather than binding *fimS* itself (15, 28). Interestingly, RNA-seq analysis did not identify differential regulation of *fimB/E* expression. To validate this, qRT-PCR analysis found that Δ*yhaJ*^*UPEC*^ did not have any impact on expression of the FimE recombinase, which favors phase ON-to-OFF switching (similar to a downshift in *fimA* expression such as that observed for Δ*yhaJ*^*UPEC*^), thereby supporting the notion of a direct influence of YhaJ on *fimS* phase switching (*SI Appendix*, Fig. S15). To test this experimentally, we used a classical PCR digestion assay that determines the ratio of *fimS* phase ON versus OFF in a cell population. DNA of *fimS* and its flanks including the inverted-repeat sequences that facilitate phase inversion was amplified from UPEC cells grown under T1F-inducing conditions (*SI Appendix*, Fig. S16*A*). Digestion of this PCR product with *Hinf*I resulted in pairwise combinations of product sizes depending on the orientation of *fimS*. WT UPEC expressed ∼50% phase ON (74/485-bp bands) whereas the Δ*yhaJ*^*UPEC*^ population shifted to only 25% phase ON and the remainder phase OFF (202/357-bp bands). Complementation of YhaJ restored phase switching toward the ON orientation in excess of the WT, resulting in 75% of the population expressing *fimS* phase ON (Fig. 5*D*). To control for these results, DNA isolated from WT EHEC contained only 100% phase OFF *fimS*, as expected. Equivalent molecular analyses of K-12 *fimS* revealed that while YhaJ can indeed bind this region in nonpathogenic *E. coli*, it is not required for normal phase switching from OFF to ON in this genetic background, mimicking the FimA expression analysis discussed above (*SI Appendix*, Fig. S16 *B* and *C*). These data collectively suggest that direct binding of YhaJ exclusively to phase OFF *fimS* facilitates inversion of the promoter element and therefore enhanced expression of T1F in pathogenic UPEC, defining a mechanism of virulence gene regulation in this pathotype.

### High Conservation of YhaJ Is Widespread among Gram-Negative Pathogens.

**Fig. 6.**
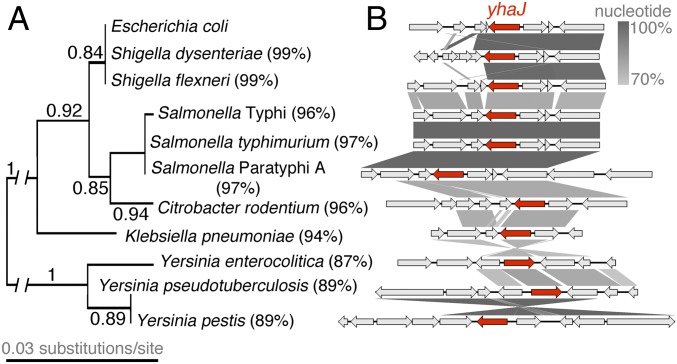
YhaJ is highly conserved among prototypical gram-negative pathogens. (*A*) Maximum-likelihood tree generated from YhaJ-coding sequences of the prototypical gram-negative pathogens with the associated branch bootstrap fractions indicated. The percentage identity to the EHEC sequence as determined by BLASTp is indicated to the right of each species name. (*B*) Genomic context of *yhaJ* from the aforementioned isolates. The *yhaJ* ORF is indicated in red and all flanking genes are in gray. The gradient between schematics represents the percentage sequence similarity as indicated on the scale bar. Genes with no connecting gradient bar are unique to the context of each isolate.

We previously used a comparative genomics analysis of 1,581 *E. coli* isolates to investigate the prevalence of *yhaJ* carriage within the species (20). We found that *yhaJ* was highly conserved, being present in 98.4% of *E. coli* (and the closely related *Shigella*) genomes. Given the distinct mechanisms uncovered in this study for YhaJ within a single species, we explored the presence of *yhaJ* among other bacterial pathogens. Using the same criteria as previously (presence being defined as a BLASTp score of greater than 70% sequence identity over at least 80% of the protein-coding region), we searched for carriage of *yhaJ* in several distinct prototypical pathogen genomes. Strikingly, this analysis identified high conservation of the *yhaJ*-coding sequence in the genomes from mechanistically and ecologically distinct gram-negative *Salmonella*, *Shigella*, *Citrobacter*, *Yersinia*, and *Klebsiella* species. Identity of the coding sequence ranged from 99% among *Salmonella* and *Shigella* isolates to 87% in *Yersinia enterocolitica* across 100% of the protein-coding sequence, with amino acid substitutions being largely clustered in the substrate-binding domain and complete conservation of the HTH at the N terminus (Fig. 6*A*). Furthermore, the genomic context of *yhaJ* displayed varying levels of heterogeneity (Fig. 6*B*). Carriage of *yhaJ* was always accompanied by the divergently transcribed *yhaK* but genes flanking this pairing were not always conserved, with the presence of insertion sequences and strain-specific genes directly adjacent to *yhaJ/K*, particularly for *Shigella dysenteriae*, *Citrobacter rodentium*, *Klebsiella pneumoniae*, and *Yersinia pseudotuberculosis*. Despite these differences, the high conservation of YhaJ itself suggests that this transcription factor likely plays important regulatory roles tailored to each individual pathogen, much like the intraspecies mechanisms described here for *E. coli*. Indeed, our study focused on 2 prototypical EHEC and UPEC isolates but, in reality, even these pathotypes will exhibit extensive genetic diversity, and thus possible refinements in transcription factor behavior depending on the genetic background. We hypothesize that recycling of this core transcription factor will be widespread among several pathogens that occupy distinct ecological niches, facilitating regulation of unique virulence mechanisms, and we are currently investigating this postulate.

## Discussion

Gene-regulatory architecture is the baseline for how a bacterial species exhibits a defined lifestyle and therefore occupies a distinct niche. However, given the mosaic nature of bacterial genomes, whereby the gene content of an individual can vary dramatically as a result of horizontal gene transfer and genome minimization, gene-regulatory architecture must be adapted to cope with such variation (4, 5). Personalized regulation is a hallmark of bacterial pathogens that need to tailor their gene expression with a focus on virulence factors, thus maximizing competitiveness within their distinct niche. Here, we have explored this concept and discovered that the highly conserved LTTR YhaJ shares few common binding sites on the chromosomes of the distinct pathotypes EHEC and UPEC, and surprisingly regulates the expression of unique targets in both genetic backgrounds, including distinct virulence genes. In EHEC, YhaJ activates the T3SS and the essential effector *nleA*, demonstrating adaptation to control multiple horizontally acquired genomic elements. In UPEC, YhaJ binds directly to the phase-variable *fimS* element, enhancing promoter inversion and thus driving T1F expression. Therefore, YhaJ acts as a key regulator of several *E. coli* virulence mechanisms related to niche-specific pathotypes.

Despite both facilitating attachment to host cells, the regulation of mechanisms driving the T3SS and T1F are mutually exclusive. Transcription of the LEE T3SS is tightly controlled by interplay between the Ler master regulator encoded on the first open reading frame (ORF) of the LEE and the xenogenic silencing protein H-NS (29, 30). H-NS represses expression of horizontally acquired DNA, such as the LEE (31). This repression is counteracted by Ler, thus driving expression of the T3SS genes (32). We previously provided evidence that YhaJ directly regulates LEE expression by binding the *ler* promoter region in vitro (20). Indeed, our ChIP-seq data identified a weak peak signal at this region but it was not statistically significant. It is likely that this is due to the competition with H-NS binding at this region, which has been found previously to mask binding sites for the FNR transcription factor in vivo (33). Our analysis did, however, identify a strong YhaJ binding site upstream of the *nleA* gene encoded on cryptic prophage 933-P (23, 34, 35). EHEC encodes over 30 NLEs that must also be expressed in sync with the needs of the cell, but the mechanisms driving NLE regulation are less clear (11, 36). We recently reported that a subset of NLEs (including *nleA*) were induced in vivo through a coordinated response to host colonization in a murine model of EHEC infection (using *C. rodentium*) (37). Indeed, *nleA* is under the control of Ler/H-NS regulation despite being encoded on a unique genetic element (38). Moreover, our in vivo transcriptome data identified expression of *yhaJ* during peak colonization of the host, collectively suggesting that YhaJ is a key regulator involved in appropriately coordinating the expression of both the LEE and *nleA* to maximize virulence.

In contrast, T1F in UPEC are controlled by a phase-variable inversion of the *fimS* promoter element (16). Regulation of T1F phase variation is a complex process largely driven indirectly by the activity of several transcription factors (including H-NS) on expression of recombinases, which in turn leads to promoter inversion (15). Strikingly, our data have revealed that YhaJ regulates T1F by directly binding to *fimS* in an orientation-specific manner and driving a phase ON bias. Aside from H-NS, 2 other nucleoid-structuring proteins (Lrp and IHF) directly bind *fimS* and are essential for its function (39, 40). Sequence analysis of *fimS* and its associated transcription factor binding sites showed that the precise YhaJ-binding motif identified by footprinting overlapped directly with the second Lrp site (*SI Appendix*, Fig. S17). *fimS* contains 3 Lrp binding sites that facilitate DNA bending to allow correct positioning of the inverted-repeat sequences for recombination and phase variation, implying that YhaJ binding at the Lrp–*fimS* interface promotes T1F phase switching directly by impacting the structure of the DNA element, thus promoting UPEC virulence gene expression (41). Furthermore, the physiological relevance of this regulation is bolstered by the up-regulation of *yhaJ* expression reported for several UPEC clinical isolates during human urinary tract infection (42). It is unclear exactly why binding occurs exclusively in the OFF orientation, perhaps requiring interaction between multiple YhaJ units and downstream sequence flanking the 3′ end of *fimS* that we did not resolve by footprinting. LTTRs typically bind canonical promoter regions as pairs of interacting dimers (21). Indeed, the concentration-dependent increase in the intensity and severity of DNA retardation observed in our EMSA analysis of YhaJ binding *fimS* is indicative of multiple occupancies bound to the fragment. Our data suggest that YhaJ functions in a nucleoid structuring-like manner, thus binding *fimS* in the OFF orientation for a specific purpose. We hypothesize that a yet-unidentified cofactor (or lack thereof) mediates this phase OFF-dependent binding, priming YhaJ for regulation of *fimS* and subsequently resulting in release of the regulator from the element once T1F expression is initiated.

**Fig. 7.**
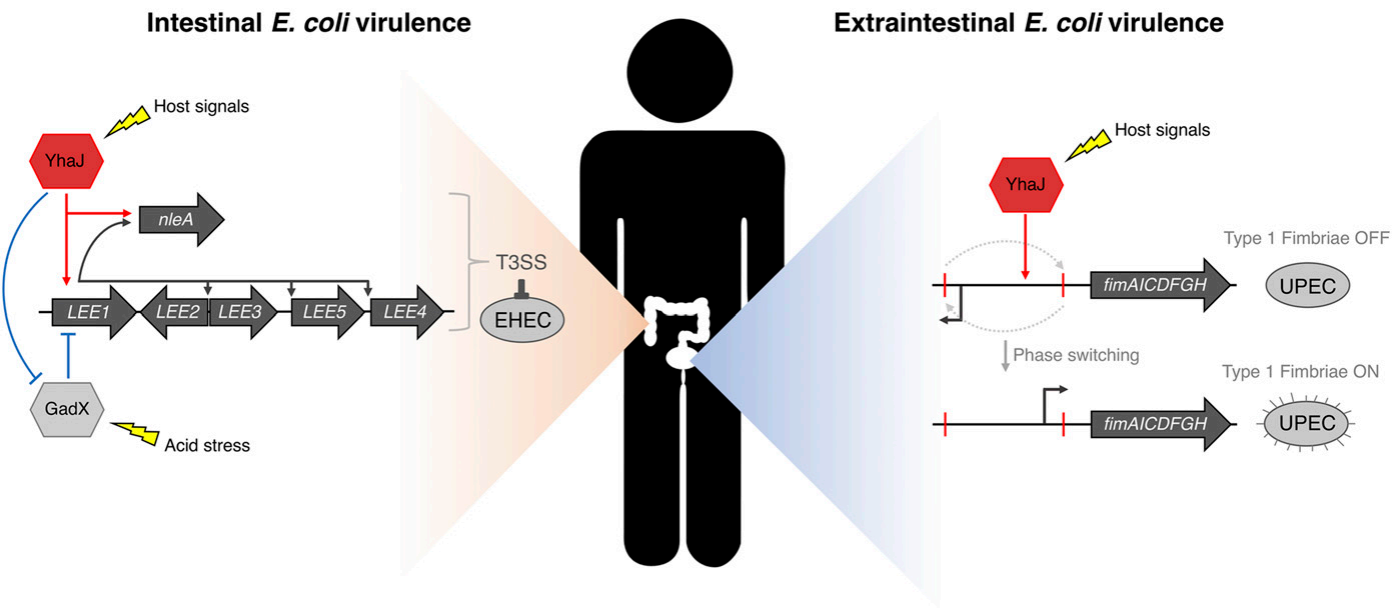
Model of pleiotropic virulence regulation by YhaJ in distinct pathotypes. EHEC (intestinal niche) virulence is controlled by YhaJ pleiotropically—direct activation of the T3SS and prophage-encoded NleA, and indirect enhancement of T3SS by direct repression of GadX. UPEC (extraintestinal niche) virulence is enhanced by YhaJ through directly promoting phase ON orientation of the Fim switch element, leading to T1F expression.

These 2 mechanisms represent highly specialized regulatory adaptations for EHEC and UPEC, given that the T3SS and T1F are instrumental for niche specification of both pathotypes (Fig. 7). Indeed, the genetic regulation of both systems is governed by defined responses to signals and cues encountered within the host (9, 15). For instance, the LEE is influenced largely by the nutritional status of the gut, host hormones, and quorum sensing, all of which coordinate T3SS expression to specify colonization of the large bowel (9, 10). T1F are similarly regulated by environmental factors of the intestine such as temperature and nutrients, but critically are also responsive to UPEC niche-specific signals such as urine and osmolality helping to refine its regulation specifically (43, 44). A common factor reported to regulate both the T3SS and T1F is pH. As it represents a crucial component of the bacterium’s lifestyle, *E. coli* are adept at responding to low pH during passage through the stomach by coupling proton consumption during glutamate decarboxylation followed by efflux of the reaction byproduct GABA (45). GadX is a global regulator that controls the response to acid but also is an indirect repressor of the T3SS (46). This is particularly relevant in light of the data presented here as YhaJ controls the T3SS both directly and indirectly via *gadX* repression. Why would this regulation be beneficial to intestinal EHEC and not extraintestinal UPEC? Virulence regulation in response to acid is specifically blocked in *E. coli* that employ T3SSs (47). However, UPEC isolates do not encode a T3SS and so recruitment of YhaJ for this purpose may be redundant (48). Importantly, YhaJ–GadX interplay occurs in the absence of pH bias in the media, suggesting that it plays a constitutive role in enhancing EHEC virulence. LTTRs classically bind small molecules in order to regulate gene expression (21). The exact ligand for YhaJ is currently unknown but nonetheless our work demonstrates that this protein plays distinct roles in transcription activation/repression without the provision of a defined exogenous ligand.

*E. coli* is an incredibly diverse species. As an example, genome comparisons of EHEC, UPEC, and K-12 revealed that only 39.2% of nonredundant protein-coding sequences were conserved between the strains, driven largely by variation in horizontal gene transfer (49). Alternatively, the concept of genome minimization suggests that occupation of a constant host environment can be associated with a loss of regulators and associated networks (4, 5). This is clearly not the case for *E. coli*, which is capable of colonizing distinct niches both within and beyond the intestine. Therefore, repurposing of highly conserved genes within the core genome, which are otherwise nonessential for survival, presents an intuitive way for *E. coli* to tailor its transcriptional architecture for performing the most important roles that the individual requires to thrive. It has been well-described that orthologous regulators can play vastly different roles between species, with perhaps the best example being PhoP that shares the regulation of only 30% of homologous gene targets between *Salmonella* and *Yersinia* (50). Furthermore, given the nature of YhaJ conservation in several gram-negative pathogens that exhibit unique lifestyles and encode distinct virulence factors such as alternative T3SSs, we anticipate this repurposing to be widespread beyond *E. coli* as a species (51). The extent of transcriptional rewiring within a single species is still up for debate but is just as critical, considering the distinct niches that can be occupied by highly virulent *E. coli* pathotypes. Our work suggests that this repurposing process may benefit the individual’s lifestyle, manifesting in pleiotropic mechanisms of virulence gene regulation by a single conserved transcription factor.

## Materials and Methods

A full list of bacterial strains, plasmids, and primers used can be found in *SI Appendix*, Tables S5–S7. A detailed description of all methodology and associated references are in *SI Appendix*, *Materials and Methods*. This includes genetic engineering of strains, cloning, growth conditions, GFP reporter assays, qRT-PCR, RNA-seq, ChIP-seq, immunoblotting, EMSA, DNaseI footprinting, microscopy, bioinformatics, and all data analysis methods and tools. All raw sequence data have been deposited in the European Nucleotide Archive and are available under accession no. PRJEB12065.

## Supplementary Material

Supplementary File
