# Supplementary material for: Distinct intraspecies virulence mechanisms regulated by a conserved transcription factor
Source: Proc Natl Acad Sci U S A. 2019 Sep 9;116(39):19695–704. doi: 10.1073/pnas.1903461116 (PMC6765310; doi:10.1073/pnas.1903461116)
Supplement: Supplementary File [file pnas.1903461116.sapp.pdf]

Supplementary Information Appendix

Distinct intra-species virulence mechanisms regulated by a conserved transcription factor

James PR Connolly, Nicky O'Boyle, Natasha CA Turner, Douglas F Browning and Andrew J Roe

Correspondence: James PR Connolly and Andrew J Roe

This PDF includes:

Materials and Methods

Figures S1 - S18

Tables S1 – S8

Supplementary references

www.pnas.org/cgi/doi/10.1073/pnas.1903461116

Materials and Methods

Bacterial growth conditions

For bacterial culture, single colonies from pure stocks were first cultured overnight in 5 ml of LB broth at 37°C before being subcultured 1/100 into minimal media (MEM-HEPES) or LB. Growth was monitored by measuring the OD_{600} of the culture. Arabinose and IPTG were supplemented to the media where specified. Antibiotics were used where appropriate at the following concentrations: 100 µg/ml ampicillin, 50 µg/ml kanamycin and 15 µg/ml chloramphenicol. All chemicals were purchased from Sigma Aldrich.

Bacterial strains, chromosomal engineering and plasmid cloning

The prototypical EHEC TUV93-0, UPEC CFT073 and K-12 MG1655 strains were used throughout this study (1–3). Mutant strains containing defined gene deletions were generated using the Lambda Red recombineering method (4). Briefly, FRT-kanamycin or FRT-chloramphenicol cassettes were amplified from pKD4 or pKD3 respectively using primers flanked by 50 bp overhangs directly homologous to the DNA sequence directly upstream or downstream of the gene of interest. This PCR product was purified by standard phenol:chloroform extraction, DpnI treated and concentration be ethanol precipitation. Strains carrying pKD46 were cultured in SOB media (ampicillin; 30°C) with 10 mM arabinose to an OD_{600} of 0.4 before being made electrocompetent by ice water washing and transformed with 2 µg of the above PCR product. Recovery was carried out on LB-kanamycin/chloramphenicol at 42°C to eliminate pKD46 and select for successful recombinants. Colonies were screened by PCR to identify successful mutants. Resistance cassettes were finally removed by transforming mutant strains with pCP20 (ampicillin; 30°C)

followed by subculturing non-selectively at 42°C on solid LB. Removal of the cassettes was screened phenotypically and confirmed by PCR.

Chromosomal tagging of YhaJ with 3X FLAG was performed using a modified Lambda Red method. The template plasmid pDOC-F was used to amplify the FLAG tag succeeded by the FRT-kanamycin cassette by PCR (5). The primers for this reaction were flanked by 50 bp of DNA directly homologous to the sequence found either side of the natural *yhaJ* stop codon. The recombination was subsequently performed and screened as above. The resulting strains contain the in-frame 3X FLAG tag fused to the YhaJ coding sequence ($YhaJ^{FLAG}$). All bacterial strains are listed in Table S5.

Complementation plasmids were generated by amplifying the gene of interest using primers flanked with *Nhe*I/*Hind*III and cloning into pBAD18 using standard digestion and ligation (6). Promoter regions to be analysed by DNAseI footprinting were amplified by PCR using primers flanked with *EcoR*I/*Hind*III and cloned into pSR (7). All clones were confirmed by sequencing (Eurofins). All restriction enzymes were purchased from New England Biolabs. Antarctic Phosphatase and T4 DNA ligase were purchased from Invitrogen. All plasmids are described in Table S6 and all primers used throughout are listed in Table S7.

Total RNA isolation and mRNA enrichment

Total RNA was obtained from bacterial cultures grown in MEM-HEPES to an OD_{600} of 0.7. 10 ml of cells were immediately fixed in RNAprotect (Qiagen) before being processed using a PureLink RNA mini kit (Ambion) to extract total RNA, according to the manufacturers specifications. Genomic DNA was removed using a TURBO DNase kit (Ambion) and samples were tested for removal using PCR. Enrichment of mRNA prior to transcriptome analysis was carried out using a MICROBExpress kit (Ambion). RNA was quantified before and after

enrichment on a LabChip GX (PerkinElmer) in order to assess the integrity of each sample. Samples were prepared in biological triplicate.

Chromatin immunoprecipitation (ChIP)

ChIP was carried out as described previously with some modifications (8). Duplicate 40 ml cultures of $EHEC^{FLAG}$ or $UPEC^{FLAG}$ were grown to an OD_{600} of 0.7 in MEM-HEPES. Wild type untagged cells were prepared identically and used as ChIP controls. Cells were crosslinked with 1% formaldehyde for 20 minutes before quenching the reaction with 0.5 M glycine. Cells were then centrifuged and washed once with1 volume of TBS before resuspending in 1 ml FA lysis buffer (50 mM Hepes-KOH, pH 7, 150 mM NaCl, 1 mM EDTA, 1% Triton X- 100, 0.1% sodium deoxycholate, 0.1% SDS) containing 4 mg/ml lysozyme and incubated for 30 minutes at 37 °C. Samples were chilled on ice and sonicated for 2 x15 cycle runs (30 seconds on/off) using a Bioruptor Pico (Diagenode). Samples were centrifuged twice at maximum speed for 5 minutes and the supernatant ("chromatin") removed. The remainder of the lysate was saved as input for ChIP-PCR. For immunoprecipitation, a 50 % slurry of Protein A sepharose beads (GE Healthcare) in TBS was pre-incubated overnight with 1 % to block. The beads were washed and resuspended as a 50 % slurry in TBS plus 0.1 % BSA. The 800 µl lysate was rotated with 60 µl of blocked beads for 2 hours at 4°C before being removed by centrifugation at 4000 rpm and replaced with a fresh 60 µl blocked beads. 4 µl of anti-FLAG antibody was added to each lysate and the tubes gently rotated overnight at 4°C. The samples were centrifuged for 1 minute at 4000 rpm and lysates carefully removed before resuspending the beads in 750 µl FA lysis buffer and transferring to a Spin-X column (Corning). Columns were rotated gently for 3 minutes before centrifugation and removal of the supernatant. 3 washes were next performed with 700 µl FA lysis buffer (the third wash containing 500 mM NaCl), followed by

one wash in ChIP wash buffer (10 mM Tris-HCl, pH 8.0, 250 mM LiCl, 1 mM EDTA, 0.5% Nonidet-P40, 0.5% sodium deoxycholate) and TE buffer (10 mM Tris-HCl, pH 7.5, 1 mM EDTA). The columns were finally transferred to a fresh dolphin-nosed tube and 100 µl of ChIP elution buffer (50 mM Tris-HCl, pH 7.5, 10 mM EDTA, 1% SDS) was added followed by incubating for 10 minutes at 65 °C with gentle agitation. Samples were eluted by centrifugation at 4000 rpm for 1 minute. Supernatants were de-crosslinked by boiling for 10 minutes and concentrated by standard phenol:chloroform extraction followed by ethanol precipitation containing 2 µl of GlycoBlue co-precipitant (Ambion). ChIP DNA was resuspended in 12 µl of nuclease free water.

Illumina library preparation and RNA/ChIP-sequencing

Library preparation of mRNA samples for RNA-seq was prepared using a TruSeq Stranded mRNA library prep kit (Illumina) according to the manufacturer's instructions. Libraries for ChIP-seq were prepared using a QiaSeq Ultralow input library prep kit (Qiagen). Sequencing was performed on the Illumina NextSeq 500 platform (75 bp length; single-end). Library generation, optimisation of amplification and sequencing were performed at the University of Glasgow Polyomics facility. Quality control of sequencing data was performed using FastQC (Babraham Bioinformatics) to assess the minimum Phred threshold of 20 and potential data contamination. The raw data has been deposited to the European Nucleotide Archive under the study accession PRJEB12065.

RNA-sequencing read mapping and differential expression analysis

Raw fastq files were imported into CLC Genomics Workbench for mapping to either the EDL933 or CFT073 reference genomes (NC_002655 and NC_004431). Reference genomes

have been previously described and were downloaded from GenBank at NCBI (https://www.ncbi.nlm.nih.gov/genbank/). Read mapping was performed using a mismatch score of 2, insertion/deletion cost of 2 and length/similarity fraction of 0.8. To exclude bias from any residual ribosomal or transfer RNA not filtered out during enrichment, reads mapped only to unique open reading frames (ORFs) were considered for downstream analysis. ORFs with <5 reads mapped on average in both WT and mutant comparisons were not included in the analysis. Mapping statistics for RNA-seq data are listed in Table S8. Pearson correlation analysis was used to assess the accuracy of replicates (Fig S17).

Differentially expressed genes (DEGs) were identified using the empirical analysis of DGE (EdgeR) tool implemented in CLC, which normalises the libraries and calculates differential expression based on replicated read count data (9). Genes were considered differentially expressed if they displayed an absolute fold change FDR-corrected *P*-value of ≤ 0.05 (Benjamini Hochberg). 3 biological replicates were used for each wild type and *ΔyhaJ* mutant.

ChIP-seq data analysis

ChIP-seq data was analysed using CLC genomics workbench. Raw fastq files were first aligned to the EHEC or UPEC reference genomes. The ChIP-seq analysis tool was used to call peaks that were significantly enriched in FLAG-tagged samples versus untagged controls (10). The tool calls peaks based on an algorithm that learns the characteristic shape of ChIP-seq peaks (an intersecting bimodal peak trend signifying enrichment of reads on the forward and reverse strands, either side of the transcription factor binding site) based on the average genome wide enrichment data. This information is then used as an optimised filter to scan the genome for individual areas of enrichment that match the characteristic peak shape, applying a score obtained from comparing an identified peak window to the shape filter and

a p-value (< 0.01) at each location based on the fact that the peak shape score follows a standard normal cumulative distribution. Peaks were called from two biological replicates and were assessed manually to ensure that they conformed to the canonical peak shape as called by the software. Regions that did not conform or were biased by excessive noise were omitted. Peak centres were characterised based on their position relative to the nearest 5' gene end and the peak window surrounding this centre was extracted from the relevant GenBank reference genome for motif analysis using MEME version 5.0.3 (11).

Quantitative real time PCR (qRT-PCR) and ChIP-PCR

Total RNA was isolated as above and TURBO DNAse treated to remove genomic DNA. Samples were quantified using a Nanodrop (Thermo Fisher) and the RNA was normalised to 1 μg as input for reverse transcription using a SuperScript First Strand cDNA kit (Invitrogen). qRT-PCR was performed using GoTaq qPCR master mix (Promega) according to the manufacturer's specifications. qRT-PCR primer pairs were tested for efficiency (95-105 %) using serial dilutions of known quantities of genomic DNA obtained using a PureLink genomic DNA kit (Thermo Fisher). The housekeeping gene *gapA* was used as an internal calibrator gene. Reactions were performed in technical and biological triplicate using the Eco real-time PCR system (Illumina) and data were analysed by the $2^{-\Delta\Delta CT}$ method (12).

For ChIP-PCR, enrichment of target DNA was calculated as signal over background using the fold enrichment method. Fold enrichment was calculated as $2^{-\Delta CT}$ ($\Delta CT = CT^{ChIP} - CT^{Control}$), where ChIP is the FLAG-tagged sample and control is the non-FLAG tagged equivalent. A control region of the *araD* gene was used to correct for non-specific enrichment. Samples for ChIP-seq for all verified for enrichment of known YhaJ targets by ChIP-PCR prior to library generation.

YhaJ protein purification

6x Histidine-tagged YhaJ was purified as described previously (13). Briefly, *E. coli* BL21-DE3 carrying pET28-*yhaJ* were cultured in LB and induced with 1 mM IPTG for 12 hours. Cell pellets were resuspended in wash buffer (200 mM NaCl, 50 mM Tris, 40 mM Imidazole, 10% glycerol) and lysed by French press. YhaJ was purified from the lysate supernatant by immobilized metal affinity ion chromatography using a HisTrap Column and AKTA-prime (GE Healthcare) followed by size-exclusion chromatography using an AKTA-prime and a Superdex S200 column (GE Healthcare). Purity of the elution fractions was determined by SDS-PAGE and concentration determined by Nanodrop 2000 (Thermo Fisher).

Electrophoretic mobility shift assay (EMSA)

EMSA analysis was performed using the DIG Gel Shift Kit system (Roche) with minor alterations. Promoter regions of interest were amplified by PCR. DNA fragments were labelled with ddUTP-11-DIG and diluted to 0.2 ng/µl. Binding reactions were set up in 20 µl and carried out for 45 minutes at room temperature using increasing concentrations of purified YhaJ (0, 0.3, 0.6 and 1 µM). Competition reactions used a 100-fold excess of unlabelled specific competitor probe. Reactions were resolved on 6% DNA retardation gels (Invitrogen) and transferred to positively charged nylon membrane (Roche) using the NOVEX system (Thermo Fisher). Membranes were UV crosslinked, blocked and probed with AP conjugated anti-DIG antibody (1/10000) according to the manufacturer specifications before being developed on the ChemiDoc imaging system (Bio-Rad). EMSAs were repeated at least twice to confirm the results.

DNaseI footprint analysis

DNaseI footprinting experiments were performed as previously described using ^{32}P-end-labelled *Aat*II-*Hind*III fragments (14). Each 20 µl reaction contained approximately 1.35 nM of template DNA in 20 mM HEPES (pH 8.0), 5 mM $MgCl_2$, 50 mM potassium glutamate, 1 mM DTT, 500 µg ml^{-1} BSA, 25 µg ml^{-1} herring sperm DNA and increasing concentrations of purified YhaJ (0, 1.5, 3, 6 and 9 µM). Samples were analysed by electrophoresis on 6 % denaturing polyacrylamide gels and calibrated with Maxam-Gilbert 'G+A' sequencing reactions. Gels were subsequently visualised on a Bio-Rad PMI Imager with Bio-Rad Quantity One software. Experiments were performed at least twice to confirm the results. Zones of protection identified were confirmed by synthesising DNA probes to contain site-specific nucleotide replacements (Eurofins) and amplifying these mutant fragments by PCR for EMSA analysis as described above.

GFP-promoter fusion transcriptional reporter assays

Promoter-GFP fusions designed to give readout of *LEE1, nleA* and *yhaJ* gene expression were generated previously (15–17). Reporter activity was measured by culturing bacteria carrying the reporter plasmids to a desired growth phase, measuring both OD_{600} and absolute fluorescence (excitation 485 nm; emission 550 nm) then expressing expression as relative fluorescence units (absolute fluorescence divided by OD_{600}). Readings were taken in black walled clear bottom plates using a FLUOstar Optima plate reader (BMG Labtech, UK). Data were corrected for background noise by subtracting signal from bacteria carrying promoterless pAJR70. Experiments were depicted as the mean ±SEM. Statistical significance was determined by way of a Students *t*-test. Experiments were performed in biological triplicate.

SDS-PAGE and immunoblot analysis

Samples of bacterial cultures were removed in 1 ml aliquots at desired timepoints, normalised by OD_{600} and centrifuged at 4000 rpm for 10 minutes followed by resuspension in 4x LDS sample buffer (Thermo Fisher). Samples were boiled for 10 minutes before being centrifuged to remove cell debris. For FimA analysis, sample buffer was acidified with concentrated HCl prior to boiling and neutralised with NaOH after. 20 µl of lysate was loaded into each well of 4-12 % Bis-Tris NuPAGE mini gels (Thermo Fisher) before being separated at 180 volts using the Novex gel system (Thermo Fisher). Separated proteins were transferred to 0.45 µm nitrocellulose membrane (GE Healthcare) using the XCell II blot module (Thermo Fisher) at 30 volts for 1 hour. Membranes were blocked with PBST containing 5 % milk and probed with anti-FimA (1/5000), anti-FLAG (1/5000), anti-DnaK (1/5000) or anti GroEL (1/25000) primary antibody followed by polyclonal anti-Rabbit HRP-conjugated secondary antibody (Sigma). Immunoblots were developed with the SuperSignal West Pico chemiluminescent substrate (Pierce) and imaged on the ChemiDoc imaging system (Bio-Rad). Experiments were performed in triplicate and band intensity was measured using ImageJ.

fimS phase orientation assay

A PCR based approach was used to assay the percentage of a cell population in which the *fimS* switch was phase ON/OFF (18). 2 µl of bacterial culture was boiled in 8 µl of nuclease free water for 10 minutes and 2 µl of this solution was used as template PCR of the *fimS* region. The resulting PCR product was PCR purified using a PCR mini kit (Qiagen) and digested with *Hinf*I before being resolved on a 2 % agarose gel. The *fimS* switch contains a unique *Hinf*I restriction site and digestion results in a banding pattern corresponding to either phase ON

(74 bp and 485 bp) or phase OFF (202 bp and 357 bp). Relative band density was determined using ImageJ and expressed as percentage phase ON vs OFF. Experiments were performed in triplicate.

Phase contrast and Immunofluorescence microscopy

100 µl samples from bacterial cultures were mixed 1:1 with 4% paraformaldehyde for 15 minutes before being centrifuged at 4000 rpm and washing of the cell pellet twice in PBS. Cells were then resuspended in PBS containing anti-FimA antibody (1/100) and incubated for 1 hour at room temperature with gentle agitation. Cells were washed again 3 times before being probed with AlexaFluor 488 (Invitrogen) secondary antibody for 1 hour and washed a final time. 5 µl aliquots of probed cell solutions were mounted onto glass slides layered with 2 % agar, allowed to soak in and then covered with a thin coverslip. Cells were imaged using a Zeiss Axioimager M1 and Zen Pro software. Phase contrast and 488 nm channel images were captured simultaneously and overlaid post-capture. Data were analysed by counting the total number of bacteria (phase) and cells emitting signal relating to FimA (Alexa-488) from 5 random fields of view in 3 independent replicates. Orientation of the *fim*-switch as well as immunoblot of FimA expression were analysed from the same cultures to confirm the results.

Acid tolerance assay

Bacteria were cultured in MEM-HEPES to an OD_{600} of 0.7 before being subcultured 1/100 into 96 well plates containing the same media adjusted to pH 3.0 with HCl and containing 1.5 mM L-glutamate (Sigma) for 2 hours at 37°C. The cultures were then serially diluted and the number of CFU/ml was enumerated by 5 µl spot plating on solid LB. The CFU/ml of the inoculum at time = 0 was enumerated also. Percentage survival was calculated as (CFU/ml at

time = 2)/(CFU/ml at time = 0) x 100 and expressed relative to the WT survival. Experiments were performed in biological triplicate.

Bioinformatic analysis of yhaJ carriage and genomic context

The YhaJ coding sequence was extracted from the complete genomes of several prototypical gram-negative pathogens using the xbase database (19). The genomes used were *E. coli* EDL933, *C. rodentium* ICC168, *K. pneumoniae* 342, *S. typhimurium* SL1344, *S.* Typhi CT18, *S.* Paratyphi A ATCC9150, *Y. pestis* CO92, *Y. enterocolitica* 8081, *Y. pseudotuberculosis* YPIII, *S. flexneri* 2002017 and *S. dysenteriae* Sd197. BLASTp was used to compare the sequences, with high conservation being considered if YhaJ shared greater than 70 % identity over at least 80 % of the coding sequence (20). Sequences were aligned using MUSCLE version 3.8.31 and a maximum likelihood tree was constructed using PhyML version 3.1 under the WAG substitution model. YhaJ genomic regions were extracted from GenBank and genomic context alignment was performed using EasyFig version 2.1 (21).

Statistical and data analysis software

RNA-seq and ChIP-seq data analysis, coverage graphs, volcano plots and pearson correlations were performed using CLC Genomics Workbench version 7.5 and the FastQC application. Gene ontology analysis was performed using ComparativeGO (22). Sequence alignments were performed using Clustal Omega. Cloning strategies and primers were designed using MacVector version 12.5. Immunoblot and agarose gel densitometry was performed using ImageJ. GraphPad Prism version 5.0 was used to generate data charts and perform statistical analysis.

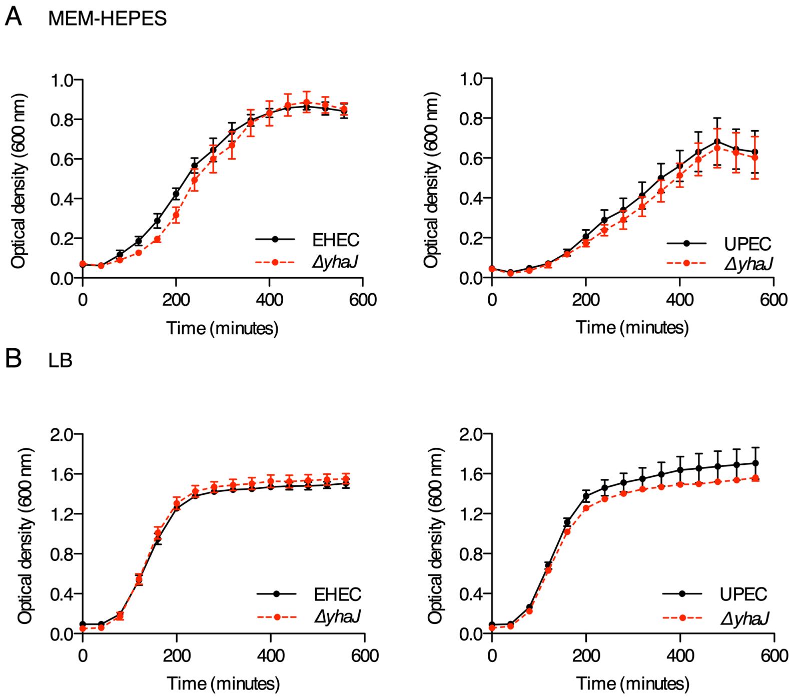

Fig. S1. YhaJ is non-essential for growth under minimal or rich conditions. (A) Growth curves of EHEC and UPEC WT (black curves) and Δ*yhaJ* (red curves) strains grown in MEM-HEPES minimal media. (B) Equivalent growth curves performed in LB rich media. Assays were performed in biological triplicate and data represents the mean OD_{600} measurements ± SD, plotted over time.

```
EHEC        MAKERALTLEALRVMDAIDRRGSFAAAADELGRVPSALSYTMQKLEEELDVVLFDRSGHR      60
UPEC        MAKERALTLEALRVMDAIDRRGSFAAAADELGRVPSALSYTMQKLEEELDVVLFDRSGHR      60
            ************************************************************

EHEC        TKFTNVGRMLLERGRVLLEAADKLTTDAEALARGWETHLTIVTEALVPTPAFFPLIDKLA      120
UPEC        TKFTNVGRMLLERGRVLLEAADKLTTDAEALARGWETHLTIVTEALVPTPAFFPLIDKLA      120
            ************************************************************

EHEC        AKANTQLAIITEVLAGAWERLEQGRADIVIAPDMHFRSSSEINSRKLYTLMNVYVAAPDH      180
UPEC        AKANTQLAIITEVLAGAWERLEQGRADIVIAPDMHFRSSSEINSRKLYTLMNVYVAAPDH      180
            ************************************************************

EHEC        PIHQEPEPLSEVTRVKYRGIAVADTARERPVLTVQLLDKQPRLTVSTIEDKRQALLAGLG      240
UPEC        PIHQEPEPLSEVTRVKYRGIAVADTARERPVLTVQLLDKQPRLTVSTIEDKRQALLAGLG      240
            ************************************************************

EHEC        VATMPYPMVEKDIAEGRLRVVSPESTSEIDIIMAWRRDSMGEAKSWCLREIPKLFNGK        298
UPEC        VATMPYPMVEKDIAEGRLRVVSPESTSEIDIIMAWRRDSMGEAKSWCLREIPKLFSGK        298
            *******************************************************.**
```

HTH-domain

Substrate binding domain

Fig. S2. Alignment of EHEC and UPEC YhaJ coding sequences. The amino acid sequences were aligned using Clustal Omega and the LTTR domain structure corresponding to the HTH domain (blue) and substrate binding domain (red) are highlighted.

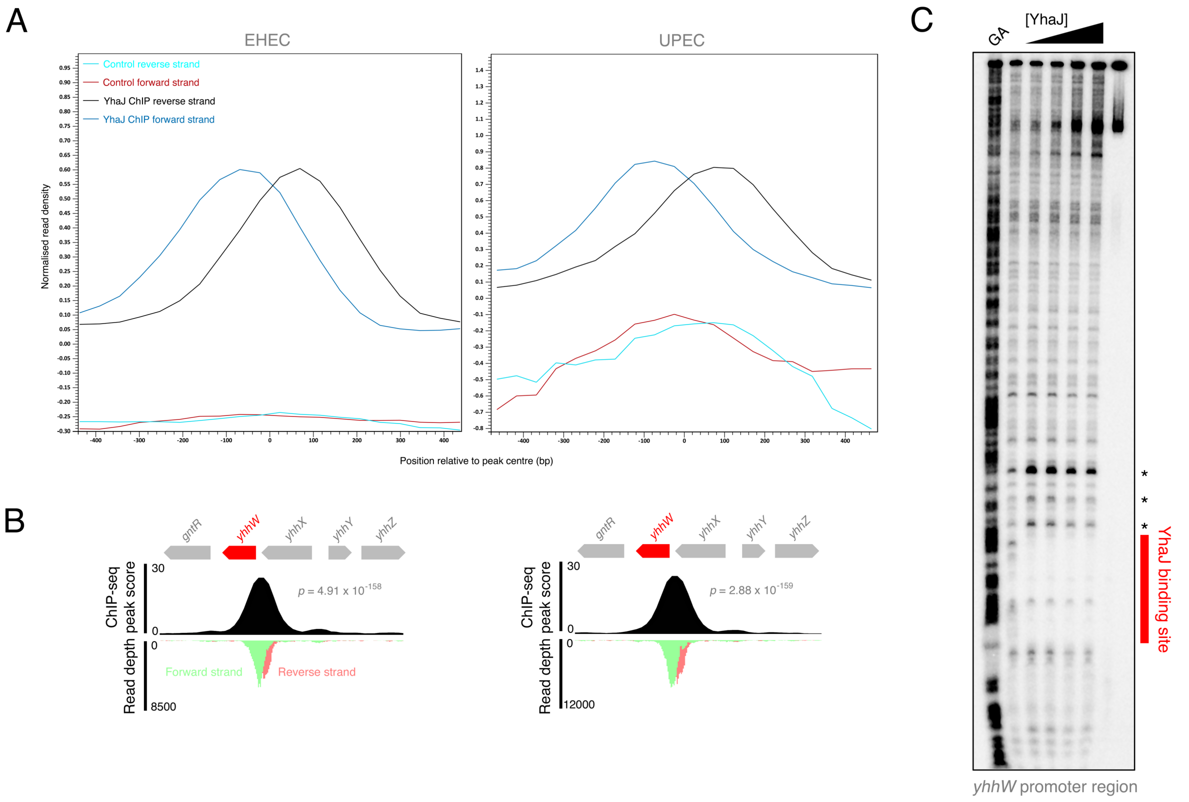

Fig. S3. Robust identification of ChIP-seq peaks for YhaJ binding site identification. (A) Peak shape filter for EHEC and UPEC ChIP-seq peak calling (biological duplicate). The blue and black traces represent the average forward and reverse reads respectively, enriched at binding sites and illustrate the canonical bimodal peak shape that genuine ChIP-seq peaks must conform to. The aqua and red traces indicate forward and reverse reads for untagged control samples. (B) Example of a high scoring ChIP-seq peak (*yhhW*) in both EHEC and UPEC. The black peak (top) represents the normalised signal enrichment peak score and the green/red peaks (bottom) illustrate raw forward/reverse read mapping, highlighting the canonical bimodal peak overlap. (C) DNAseI footprint analysis of YhaJ binding at the *yhhW* promoter region. The protected region (indicated in red) and associated hypersensitive sites correlated with the location of the ChIP-seq peak and the contained the predicted YhaJ binding motif as previously determined, thus validating the approach (23).

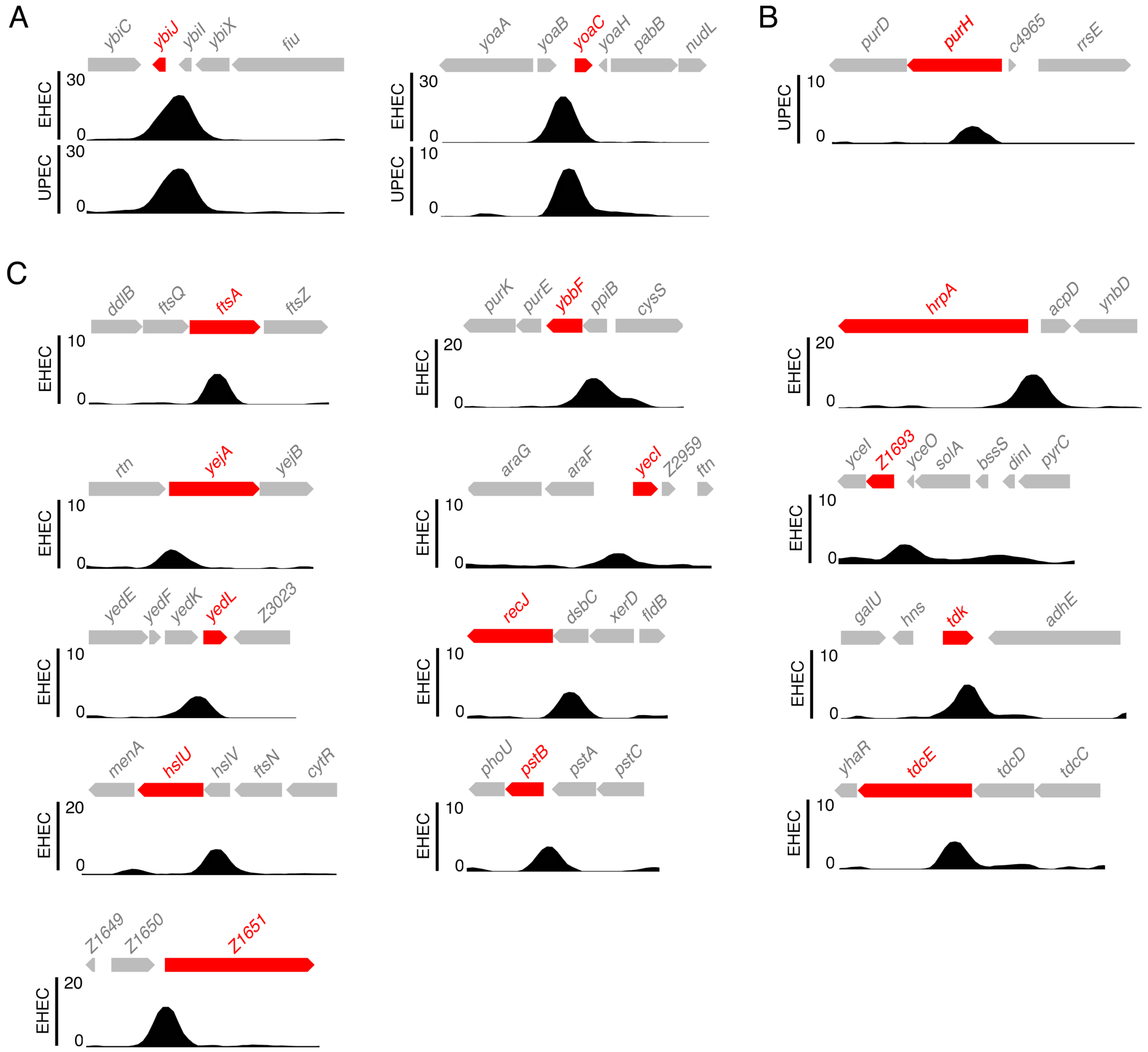

Fig. S4. Genome-wide YhaJ binding sites to conserved chromosomal regions. (A) Expanded view of ChIP-seq peaks identified for YhaJ in both EHEC and UPEC with the nearest gene 5' end highlighted in red. The scale bar represents the ChIP peak score for each binding site. (B) UPEC-specific YhaJ binding sites. (C) EHEC-specific binding sites. All ChIP-seq experiments were performed in MEM-HEPES and in biological duplicate.

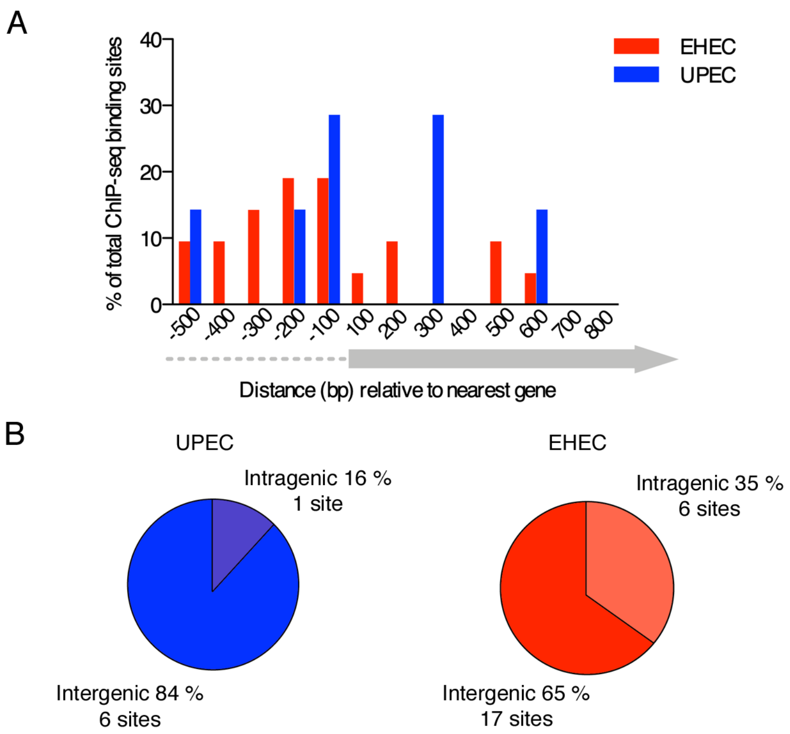

Fig. S5. Genomic context of YhaJ binding sites. (A) Positioning of YhaJ binding sites in EHEC (red) and UPEC (blue). The bars represent the percentage of binding sites relative to the nearest gene 5' end clustered into 100 bp bins. (B) Proportion of identified binding sites that are either intra or intergenic.

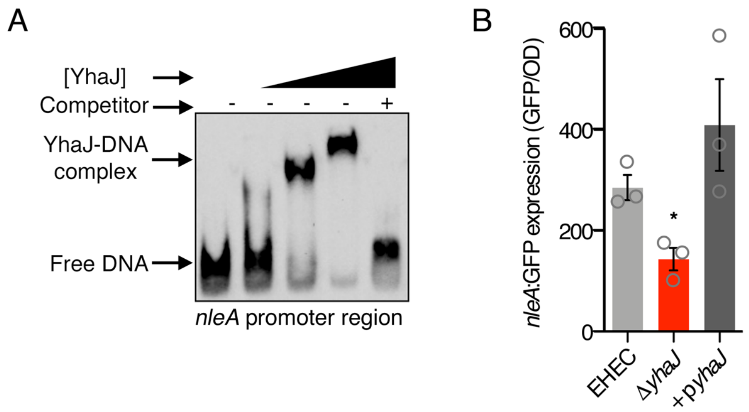

Fig. S6. YhaJ directly activates *nleA* transcription from the accessory CP-933P locus. (A) EMSA analysis confirming YhaJ binding directly to the *nleA* promoter region. Binding specificity was confirmed by the addition of excess unlabelled probe as a competitor. (B) Validation and complementation (+p*yhaJ*) of *nleA* regulation by YhaJ using an *nleA*::GFP promoter-fusion reporter. Data represents normalised fluorescence (GFP/OD_{600}) from the mean of three biological replicates ± SD. * denotes $p \leq 0.05$ (Students *t*-test).

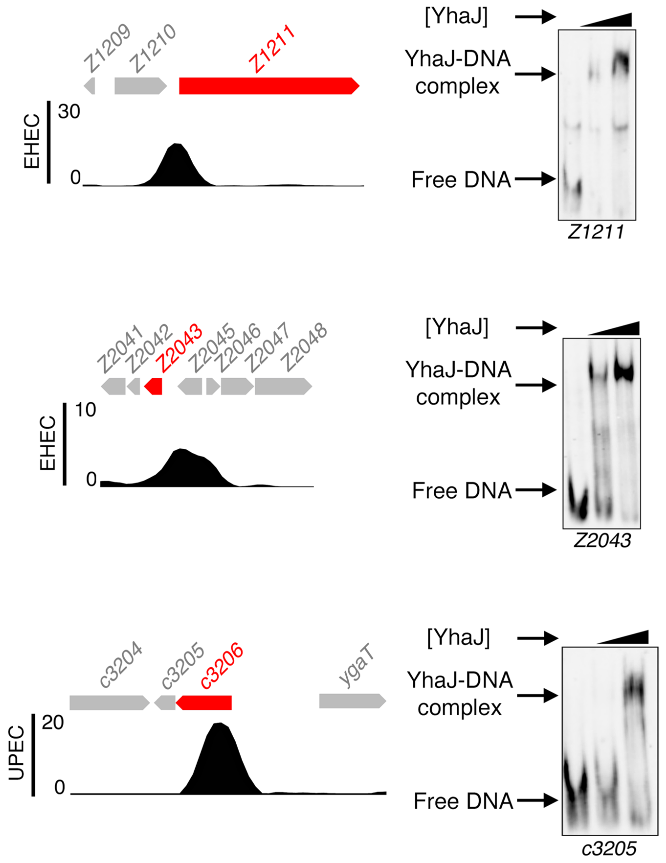

Fig. S7. YhaJ binds to pathotype-specific genomic sites. Expanded view of unique YhaJ binding sites for genes found exclusively in EHEC or UPEC. An EMSA accompanies each peak to verify the ability of YhaJ to bind these pathotype-specific sites.

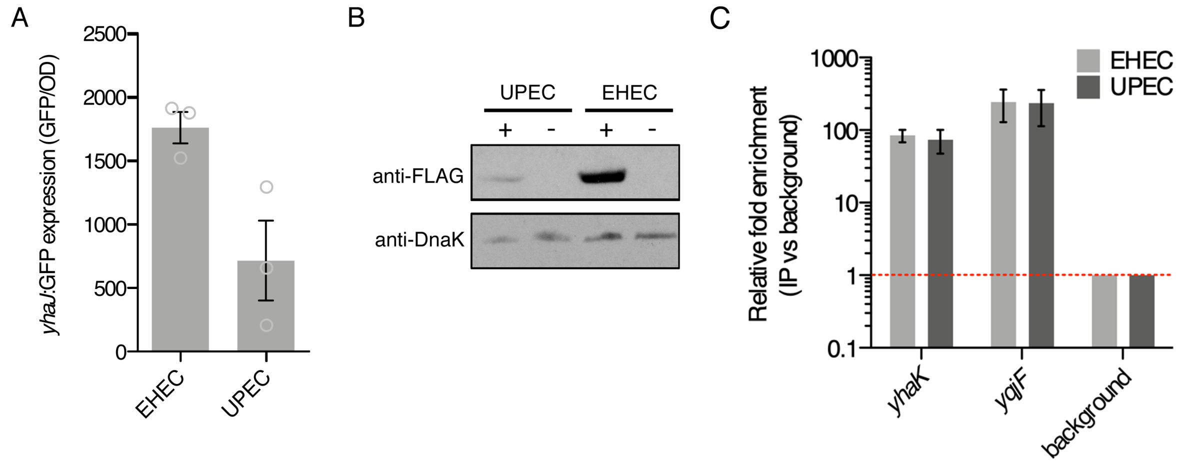

Fig. S8. YhaJ displays higher native expression in EHEC compared to UPEC. (A) Transcriptional reporter data of EHEC and UPEC cells carrying a plasmid-borne *yhaJ*::GFP promoter fusion. Cells were grown in MEM-HEPES and measurements were taken at an OD_{600} of ~0.7. Data represents the mean ± SD of three biological replicates. (B) Immunoblot analysis of WT UPEC and EHEC (-) or UPEC and EHEC encoding chromosomally FLAG-tagged YhaJ (+) grown in MEM-HEPES. Anti-FLAG antibody assessed YhaJ-FLAG native expression levels whereas anti-DnaK assessed protein loading. (C) ChIP-PCR analysis of known YhaJ targets (*yhaK* and *yqjF*) validate enrichment of regulon members versus non-specific background noise (fold-enrichment above red dotted line). Relative enrichment of the genes was assessed for EHEC and UPEC. Data represents two biological replicates.

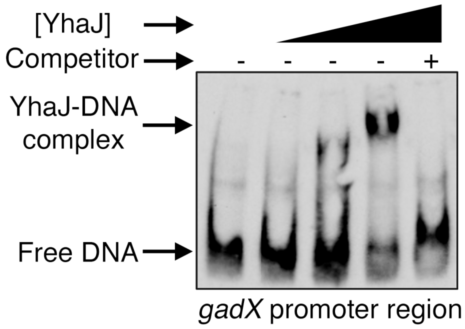

Fig. S9. YhaJ regulates *gadX* expression in EHEC. EMSA analysis confirming YhaJ binding directly upstream of *gadX.* Binding specificity was confirmed by the addition of excess unlabelled probe as a competitor.

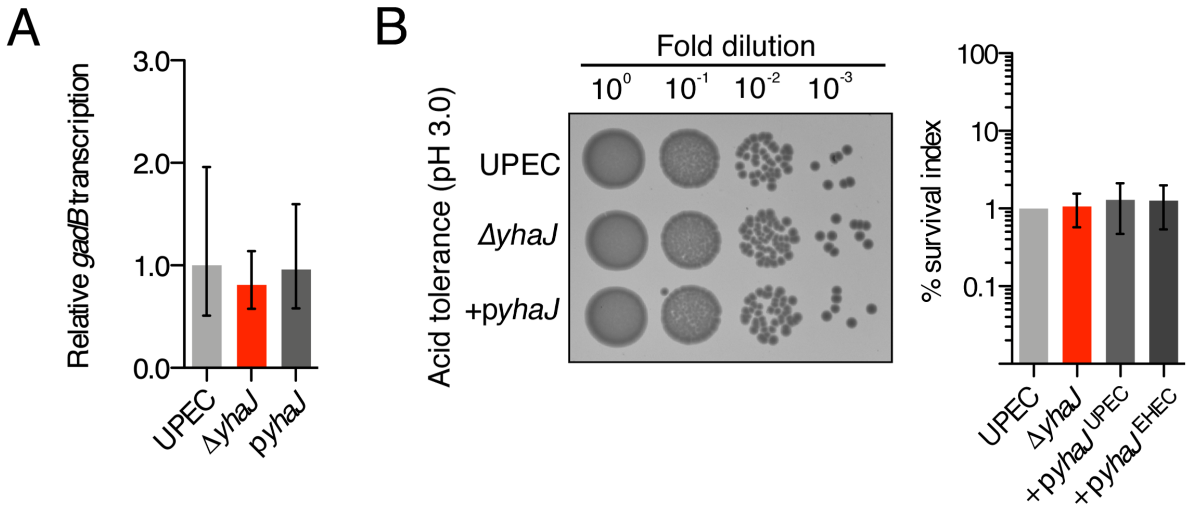

Fig. S10. YhaJ does not regulate GAD gene expression and acid tolerance in UPEC. (A) qRT-PCR analysis of relative *gadB* transcription in UPEC, Δ*yhaJ* and +p*yhaJ* genetic backgrounds. (B) Acid tolerance assay of the corresponding strains exposed to acidic minimal media and associated quantification of CFUs representing the mean survival index relative to the WT (± SD; three biological replicates). The Δ*yhaJ* strain was transformed with both UPEC and EHEC *yhaJ* alleles expressed *in trans* (+p*yhaJ*^{UPEC} and +p*yhaJ*^{EHEC} respectively).

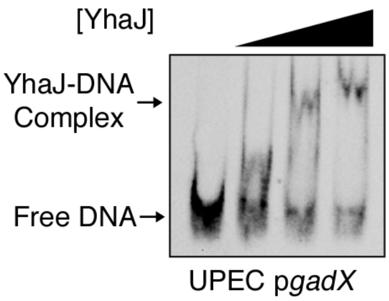

Fig. S11. YhaJ can bind to the UPEC *gadX* promoter region *in vitro.* EMSA analysis of YhaJ binding to the *gadX* promoter region confirms the ability to bind this conserved sequence despite the lack of this regulation *in vivo.* EMSAs were performed on three independent occasions to confirm the results.

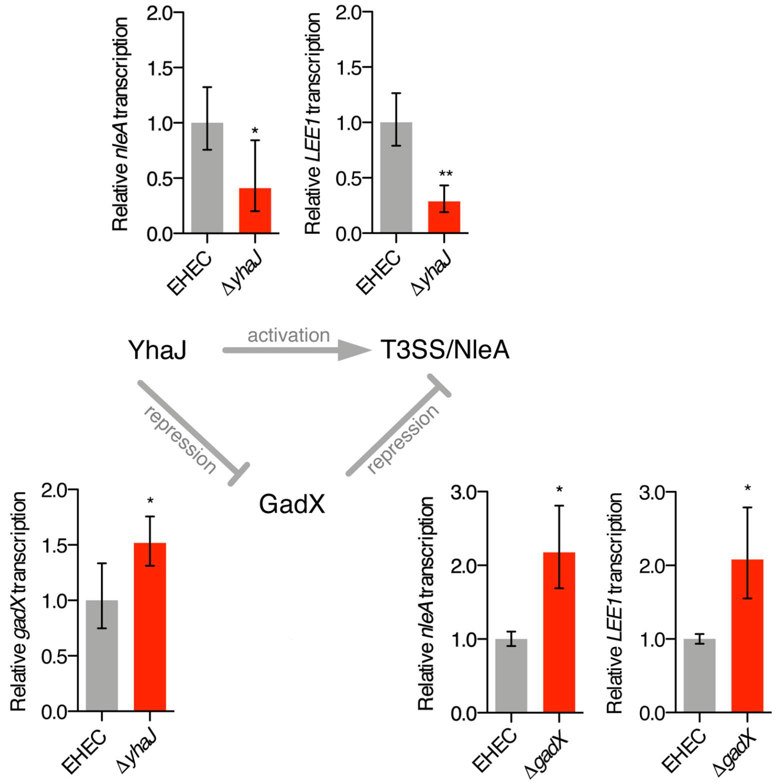

Fig. S12. YhaJ and GadX regulatory interplay in EHEC. The centre of the figure contains a diagram depicting the interplay between YhaJ and GadX regulation in EHEC. GadX represses the T3SS master regulator *ler* (encoded on *LEE1*) which in turn represses *nleA.* YhaJ activates the LEE and *nleA* directly. YhaJ represses *gadX* to block expression of acid tolerance and enhance the T3SS co-ordinately. Overlaid at each node of the diagram is qRT-PCR analysis confirming each element of this regulatory interplay. * denotes $p \leq 0.05$ derived from three biological replicates ± SD (Students *t*-test).

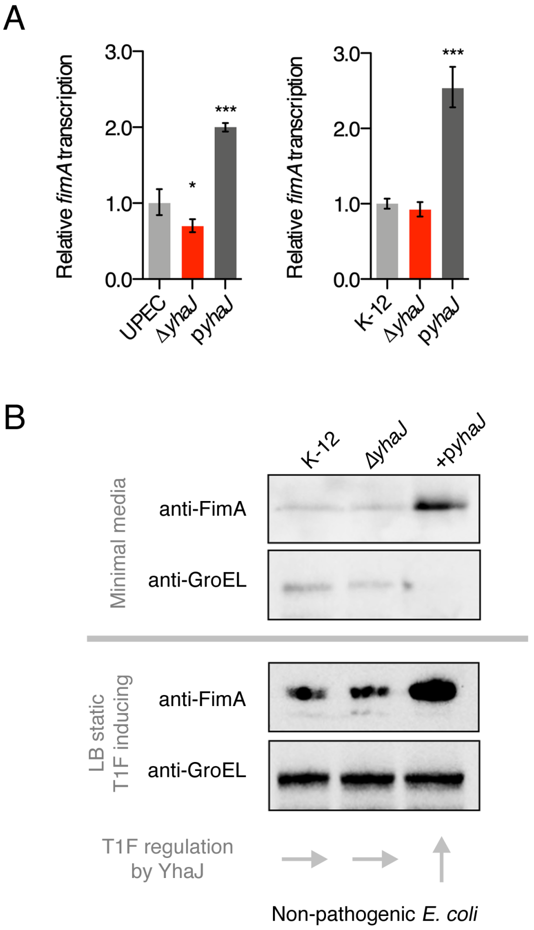

Fig. S13. YhaJ enhances but is not required for T1F expression in non-pathogenic K-12. (A) qRT-PCR analysis of relative *fimA* transcription in Δ*yhaJ* and complemented +p*yhaJ* from UPEC and K-12 genetic backgrounds. * and *** denote $p \leq 0.05$ and $p \leq 0.001$ respectively, derived from three biological replicates ± SD (Students *t*-test). (B) Immunoblot analysis of FimA levels from K-12, Δ*yhaJ* and +p*yhaJ* cell lysates grown in minimal media and under T1F-inducing conditions. The impact of YhaJ deletion or complementation of T1F expression is highlighted below. Experiments were performed in biological triplicate.

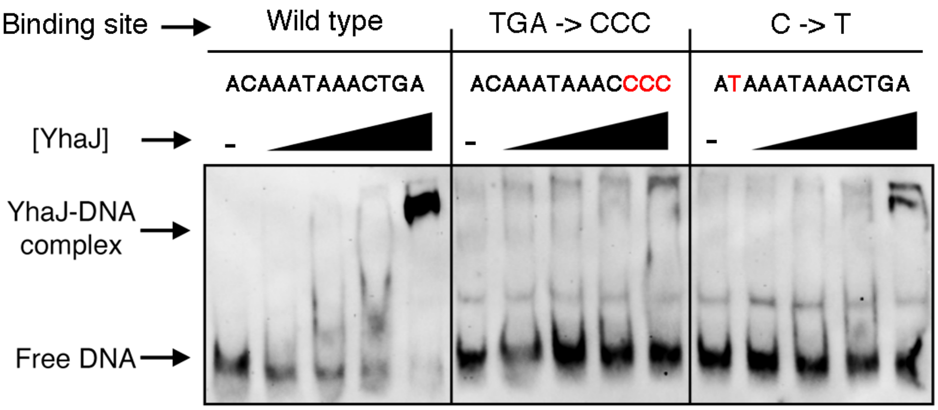

Fig. S14. Confirmation of critical YhaJ binding residues in the *fimS* site. EMSA analysis of YhaJ binding to wild type *fimS*-OFF from UPEC, and two mutant derivatives of the binding sequence. The specific nucleotide replacements are highlighted in red above each panel. EMSAs were repeated in triplicate.

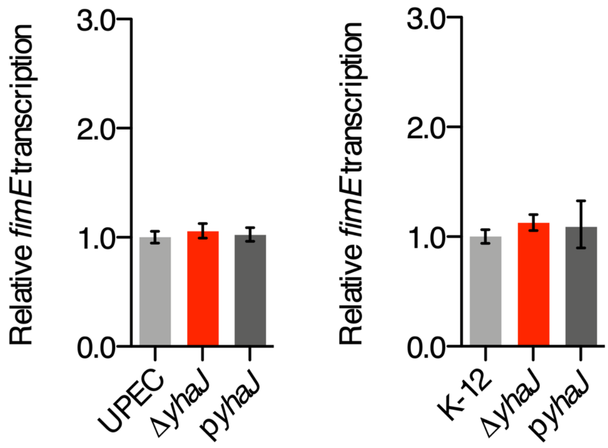

Fig. S15. YhaJ-mediated T1F phase switching is not driven via phase ON recombinase activity. qRT-PCR analysis of relative *fimE* transcription in WT UPEC, K-12 and respective Δ*yhaJ* or +p*yhaJ* genetic backgrounds. Data is derived from three biological replicates ± SD.

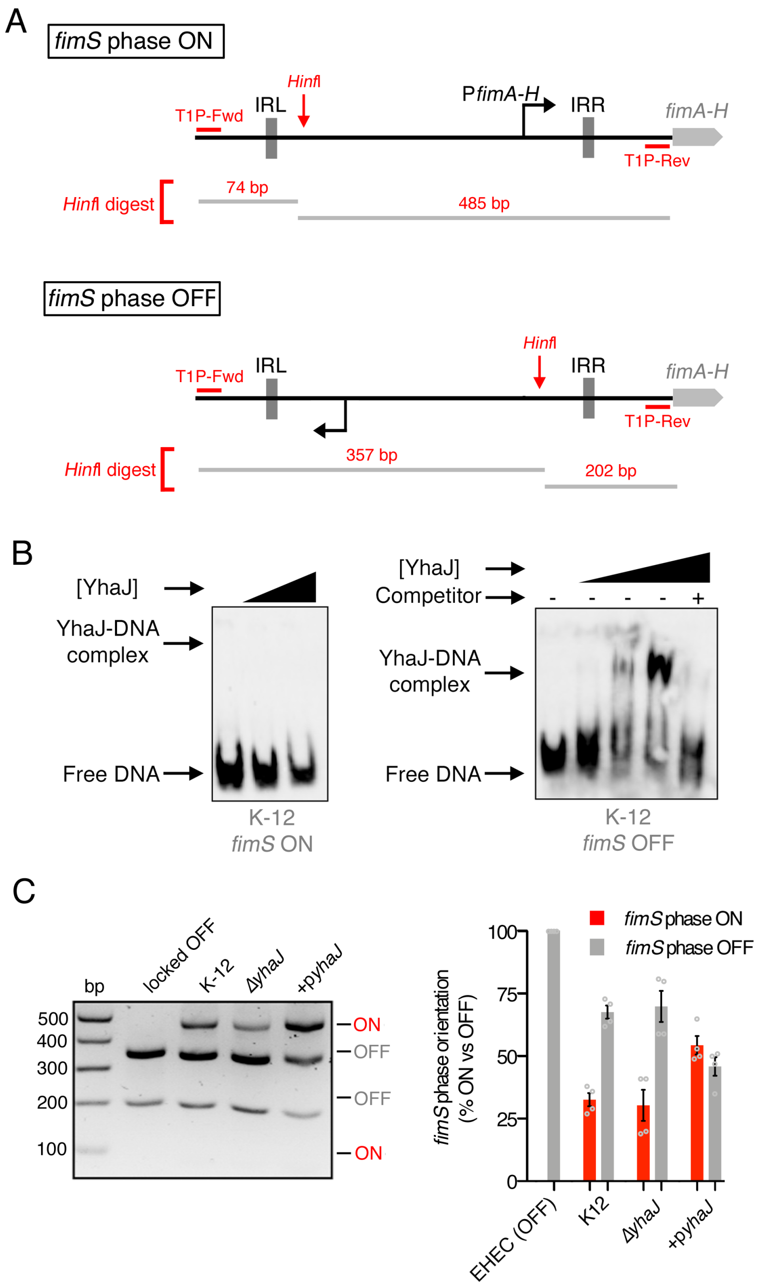

Fig. S16. YhaJ binds functionally to *fimS* in K-12 but is not required for phase switching. (A) Schematic illustration of the T1F phase assay strategy to determine levels of *fimS* phase ON or OFF in a cell population. The *fimS* region including the inverted repeat left (IRL) and right (IRR) regions was amplified from cell population by PCR (559 bp), purified and digested with *HinF*I (unique restriction site indicated by red arrow). The digestion products were resolved on 2 % agarose gel, obtaining bands of 74/485 bp for phase ON or 357/202 bp for phase OFF. (B) EMSA analysis confirming that YhaJ binds to K-12 *fimS* exclusively in the OFF orientation. The band shift specificity was confirmed by including an excess of unlabelled *fimS* DNA as a competitor. (C) T1F phase assay of K-12, Δ*yhaJ* and +p*yhaJ* cell populations resolved on 2 % agarose. Data from 4 biological replicates was quantified as % phase ON/OFF within the entire population (determined using band densitometry). WT EHEC which encodes a permanently locked OFF *fimS* was used as a control for all phase orientation experiments.

```
EHEC        TGATTTAACTTATTGATAATAAAGTTAAAAAACAAATAAATACAAGACAATTGGGGCCAA   60
UPEC        TGATTTAACTTATTGATAATAAAGTTAAAAAACAAATAAATACAAGACAATTGGGGCCAA   60
            ************************************************************

EHEC        ACTGTCCATATCATAAATAAGTTACGTATTTTTTCTCAAGCATAAAAATTTTA-------   113
UPEC        ACTGTCTATATCATAAATAAGTTACGTATTTTTTCTCAAGCATAAAAATATTAAAAAACG   120
            ****** ****************************************** ***

EHEC        ---------AACTAACTGTTTGATATGTAAATTATTTCTCTTGTAAATTAATTTCACATC   164
UPEC        ACAAAAAGCATCTAACTGTTTGATATGTAAATTATTTCTCTTGTAAATTAATTTCACATC   180
                     * *************************************************

EHEC        ACCTCCGCTATATGTAAAGCTAACGTTTCTGTGGCTCGACGCATCTTCCTCATTCTTCTC   224
UPEC        ACCTCCGCTATATGTAAAGCTAACGTTTCTGTAGCTCGACGCAACTTCCTCATTCTTCTC   240
            ******************************** ********** ****************

EHEC        TCCAAAAACCACCTCATGCAATATAAAAATCTATAAATAAAGATAACAATAGAATATTAA   284
UPEC        TCCAAAAACCACCTCATGCAATATAAAAAACTGCAAATAAAGATAACTATAGAACATTAA   300
            ***************************** **  ************* ****** *****

EHEC        TCCAGCAAATAAACTGAAAAAGTTTGTGCGCGATGCTTTCCTCTATGAGTCAAAATGGCC   344
UPEC        GCCAACAAATAAACTGAAAAAGTTTGTGCGCGATGCTTTCCTCTATGAGTCAAAATGGCC   360
             *** *******************************************************

EHEC        CCAAATGTTTCATCTTTTGGGGGAAAACTGTGCAGTGTTGGCAGTCAAACTCGTTGACAA   404
UPEC        CCAAATGTTTCATCTTTTGGGGGAAAACTGTGCAGTGTTGGCAGTCAAACTCGTTGACAA   420
            ************************************************************

EHEC        AACAAAGTGTACAGAACGACTGCCCATGTCGATTTAGAAATAGTTTTTTGAAAGGAAAGC   464
UPEC        AACAAAGTGTACAGAACGACTGCCCATGTCGATTTAGAAATAGTTTTTTTAAAGGAAAGC   480
            ************************************************* **********

EHEC        AGCATG 470
UPEC        AGCATG 486
            ******
```

-10/-35 promoter elements

Inverted repeat regions

IHF binding site

LRP binding site

YhaJ binding site

Fig. S17. YhaJ overlaps with other *fimS* regulator binding sites. Depicted is the *fimS* sequence from EHEC and UPEC illustrating all regulatory features of this genetic element (24). The 16 bp deletion in EHEC renders *fimS* permanently locked OFF. The coloured boxes illustrate the following features: Red = inverted repeat recombination sites facilitating phase variation of *fimS;* Green = the *fimA* -10/-35 promoter elements; Yellow = The accessory inverted repeats within the *fimS* and *fimA* intergenic region; Grey = IHF binding sites; Aqua = Lrp binding sites. The YhaJ binding site is underlined.

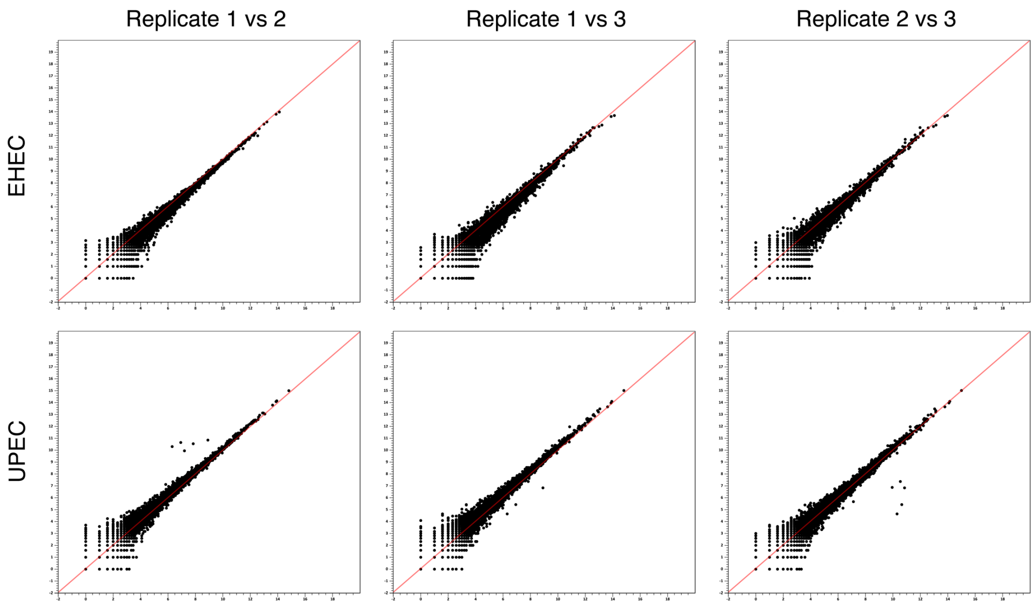

Fig. S18. Validation of RNA-seq replicates data. Pearson-Spearman plots showing pairwise comparison of biological triplicate RNA-seq data for EHEC and UPEC WT (y-axis) versus Δ*yhaJ* (x-axis). The data spread indicates gene expression values (unique counts) and the red line represents a perfect correlation of *R* = 1.

Table S1. EHEC WT versus Δ*yhaJ*^{EHEC} differentially expressed genes identified by RNA-seq.

			**EHEC WT**				**EHEC Δ*yhaJ***			
**Gene**	**FC**[1]	**FDR p-val**[2]	**Exp. val**[3]	**Exp. val**	**Exp. val**	**Means**	**Exp. val**	**Exp. val**	**Exp. val**	**Means**
yqjF	4.68	0.02	2	1	2	1.67	5	9	8	7.33
frdC	3.72	0.05	5	1	1	2.33	9	5	10	8
xasA	3.1	9.11E-11	304	300	443	349	1,308.00	645	1,021.00	991.33
gadB	3.01	6.79E-19	409	316	429	384.67	1,268.00	738	1,121.00	1,042.33
gadA	2.79	4.39E-11	178	185	228	197	633	325	543	500.33
yhiM	2.74	5.00E-05	48	24	22	31.33	114	45	70	76.33
metE	2.7	1.92E-06	446	476	691	537.67	1,626.00	752	1,633.00	1,337.00
csgB	2.68	9.77E-06	38	40	83	53.67	127	121	142	130
hdeD	2.62	1.71E-08	83	66	76	75	255	113	163	177
csgA	2.51	4.39E-11	180	144	234	186	488	364	406	419.33
hdeA	2.49	6.77E-09	443	396	576	471.67	1,375.00	742	1,085.00	1,067.33
yhiU	2.45	2.73E-05	30	23	45	32.67	83	51	82	72
yhiV	2.31	1.15E-12	90	87	96	91	219	150	193	187.33
ompC	2.03	3.01E-07	3,316.00	3,132.00	4,237.00	3,561.67	7,784.00	4,955.00	6,904.00	6,547.67
ecnB	1.91	2.96E-03	34	29	33	32	74	41	49	54.67
slp	1.88	1.61E-08	365	372	394	377	770	497	643	636.67
msyB	1.83	0.02	24	17	26	22.33	39	37	33	36.33
ybaS	1.82	8.99E-03	27	28	30	28.33	43	49	45	45.67
ydaJ	1.81	9.48E-04	117	91	101	103	186	102	213	167
yqjD	1.74	2.27E-03	50	53	63	55.33	95	65	99	86.33
dps	1.74	1.16E-08	967	951	898	938.67	1,697.00	1,126.00	1,561.00	1,461.33
katE	1.71	1.68E-03	72	57	52	60.33	112	62	102	92
lysA	1.7	5.86E-03	40	51	40	43.67	78	52	69	66.33
poxB	1.66	4.77E-04	89	83	86	86	153	93	137	127.67
gcd	1.57	4.09E-03	103	120	85	102.67	163	110	157	143.33
ybaY	1.56	5.20E-04	115	118	104	112.33	156	139	173	156
yqjC	1.55	0.03	46	44	36	42	62	54	57	57.67
gcvP	1.46	3.44E-03	138	145	137	140	191	167	187	181.67
metK	1.46	7.78E-04	532	448	530	503.33	726	533	709	656
tktB	1.45	0.01	167	157	166	163.33	264	170	201	211.67
grxB	1.44	3.80E-03	175	156	181	170.67	241	193	224	219.33
elaB	1.44	0.02	94	82	79	85	126	84	117	109
cysQ	1.41	0.05	79	76	75	76.67	104	93	91	96
arcA	1.41	9.86E-03	288	244	273	268.33	372	261	379	337.33
yagU	1.4	0.03	131	104	119	118	159	136	143	146
ycdO	1.37	0.02	509	411	495	471.67	663	460	609	577.33
cfa	1.35	0.02	315	252	271	279.33	393	269	346	336
Z3260	1.34	0.03	211	180	187	192.67	262	190	236	229.33
ompX	1.34	6.07E-03	882	791	844	839	1,089.00	837	1,075.00	1,000.33
osmC	1.33	0.03	298	246	274	272.67	365	265	335	321.67
ompA	1.31	0.03	5,327.00	4,707.00	5,471.00	5,168.33	6,391.00	5,278.00	6,494.00	6,054.33
himA	1.26	0.03	776	638	683	699	836	693	809	779.33
rseA	-1.33	0.04	550	447	356	451	357	255	284	298.67
ftsJ	-1.39	0.01	326	275	214	271.67	200	147	170	172.33
yceI	-1.44	7.80E-03	200	204	191	198.33	125	115	128	122.67
yrfE	-1.51	0.04	139	109	76	108	73	48	68	63
rpsT	-1.51	2.37E-04	453	334	315	367.33	261	173	211	215
yihE	-1.52	0.01	98	83	82	87.67	57	40	56	51
uvrY	-1.54	7.52E-03	172	121	111	134.67	97	69	66	77.33
Z4328	-1.56	5.04E-03	133	131	100	121.33	63	70	73	68.67
infA	-1.62	0.03	172	157	93	140.67	108	56	67	77
espB	-1.62	5.72E-03	15,112.00	14,149.00	12,379.00	13,880.00	5,527.00	8,154.00	8,806.00	7,495.67
yejG	-1.62	0.02	104	83	56	81	50	39	43	44
escC	-1.64	3.24E-04	725	598	454	592.33	268	319	361	316
eae	-1.64	4.40E-03	8,167.00	7,947.00	6,919.00	7,677.67	3,117.00	3,836.00	5,434.00	4,129.00
escV	-1.65	3.48E-06	416	366	300	360.67	183	171	224	192.67
espD	-1.68	2.96E-03	17,796.00	16,101.00	13,106.00	15,667.67	5,933.00	8,961.00	9,475.00	8,123.00
espA	-1.68	3.23E-03	9,493.00	9,043.00	7,513.00	8,683.00	3,249.00	4,936.00	5,317.00	4,500.67
tir	-1.7	5.65E-04	5,210.00	4,744.00	4,053.00	4,669.00	1,835.00	2,392.00	3,010.00	2,412.33
rpsU	-1.71	0.01	309	226	128	221	131	107	100	112.67
yciS	-1.71	0.02	58	53	47	52.67	36	18	28	27.33
secG	-1.73	3.48E-04	482	335	234	350.33	207	143	181	177

yljA	-1.74	0.04	63	53	38	51.33	37	21	20	26
Z1824	-1.76	1.01E-03	133	117	83	111	52	42	73	55.67
Z5104	-1.78	3.84E-04	2,292.00	2,262.00	1,895.00	2,149.67	787	1,216.00	1,165.00	1,056.00
Z5125	-1.78	1.89E-06	271	260	204	245	108	123	132	121
Z5114	-1.8	7.19E-06	175	155	135	155	67	79	82	76
stpA	-1.81	0.02	53	37	43	44.33	29	21	15	21.67
espF	-1.83	2.04E-04	937	840	698	825	289	375	521	395
Z5111	-1.84	1.55E-04	1,007.00	966	770	914.33	318	450	540	436
bax	-1.84	7.19E-06	223	201	143	189	111	65	97	91
escN	-1.85	1.92E-06	451	382	293	375.33	156	156	222	178
Z5123	-1.87	7.19E-06	397	352	273	340.67	126	160	193	159.67
Z5129	-1.9	5.05E-05	481	383	298	387.33	133	199	198	176.67
Z6024	-1.91	1.55E-04	188	141	109	146	52	78	70	66.67
pitB	-1.92	0.04	42	25	27	31.33	15	13	15	14.33
escD	-1.93	1.07E-04	299	318	168	261.67	111	119	127	119
sepL	-1.95	4.21E-05	1,366.00	1,043.00	704	1,037.67	368	472	542	460.67
nuoJ	-2.02	0.01	42	48	29	39.67	19	19	14	17.33
Z5140	-2.03	4.23E-06	373	313	208	298	105	141	136	127.33
Z0985	-2.05	0.01	45	29	26	33.33	17	13	13	14.33
Z1693	-2.06	0.04	25	25	27	25.67	10	12	11	11
Z5142	-2.07	1.87E-04	282	245	141	222.67	80	73	130	94.33
Z1074	-2.08	8.65E-03	40	35	24	33	15	12	15	14
sepQ	-2.1	9.80E-06	270	245	149	221.33	76	82	119	92.33
Z5139	-2.14	2.57E-03	94	83	45	74	24	38	28	30
Z5113	-2.14	1.38E-07	526	443	298	422.33	141	181	192	171.33
escF	-2.16	2.72E-05	311	243	164	239.33	67	105	115	95.67
Z0955	-2.17	2.47E-08	953	966	662	860.33	282	337	427	348.67
Z5117	-2.17	8.37E-03	43	34	22	33	12	13	15	13.33
cesD	-2.22	2.47E-08	248	227	144	206.33	76	87	81	81.33
Z5128	-2.23	1.74E-04	222	134	92	149.33	44	62	67	57.67
evgA	-2.23	0.04	29	21	21	23.67	11	3	14	9.33
yheA	-2.23	1.05E-04	101	77	51	76.33	41	26	23	30
Z5115	-2.25	2.69E-06	409	295	197	300.33	90	114	142	115.33
Z5137	-2.26	3.48E-08	289	235	156	226.67	75	86	102	87.67
sdhC	-2.26	6.18E-03	114	71	35	73.33	34	26	24	28
Z3072	-2.27	0.01	36	25	19	26.67	13	7	11	10.33
Z3931	-2.29	2.02E-03	48	40	27	38.33	19	16	9	14.67
Z5131	-2.36	0.01	36	32	18	28.67	16	9	7	10.67
Z2974	-2.37	0.03	28	19	18	21.67	12	8	4	8
Z5102	-2.38	1.05E-04	361	324	205	296.67	62	128	133	107.67
Z5138	-2.41	4.23E-06	155	125	68	116	40	41	45	42
Z3768	-2.41	1.81E-03	69	87	35	63.67	30	21	19	23.33
Z1823	-2.49	0.03	24	15	15	18	7	6	6	6.33
Z5187	-2.55	6.27E-03	40	28	17	28.33	11	11	7	9.67
acpD	-2.65	6.56E-06	63	40	48	50.33	18	20	12	16.67
Z3071	-2.7	3.39E-08	133	112	68	104.33	29	37	35	33.67
escJ	-2.71	2.40E-06	330	235	123	229.33	57	74	87	72.67
ylaD	-2.77	9.49E-04	35	25	19	26.33	9	9	7	8.33
Z5745	-2.78	8.88E-03	32	24	14	23.33	11	6	5	7.33
Z2005	-2.95	3.32E-03	69	62	20	50.33	15	18	11	14.67
Z5118	-2.97	4.56E-07	79	57	41	59	14	18	20	17.33
Z5143	-3.18	9.22E-05	50	36	24	36.67	6	14	10	10
Z5136	-3.18	5.93E-10	194	186	91	157	38	44	47	43
escU	-3.26	7.19E-06	64	47	32	47.67	8	18	12	12.67
escR	-3.34	7.99E-08	99	56	45	66.67	18	16	18	17.33
Z0405	-3.44	0.03	14	14	8	12	4	4	1	3
Z6025	-4.16	1.47E-04	20	19	14	17.67	4	4	3	3.67
Z0397	-4.28	2.20E-03	17	12	11	13.33	1	2	5	2.67
Z2873	-4.76	0.03	9	7	7	7.67	2	0	2	1.33
escT	-5.07	9.57E-08	68	59	26	51	10	11	5	8.67
thrL	-5.35	0.05	11	12	3	8.67	3	1	0	1.33
yhaJ	-411.13	2.35E-38	58	51	54	54.33	0	0	0	0

[1]Fold change derived from EdgeR normalised data
[2]FC-associated *p*-value with a false discovery rate (FDR) of 5% applied
[3]Expression values depicted as unique reads mapped per gene

Table S2. UPEC WT versus Δ*yhaJ*^{UPEC} differentially expressed genes identified by RNA-seq.

			**UPEC WT**				**UPEC Δ*yhaJ***			
**Gene**	**FC[1]**	**FDR p-val[2]**	**Exp. val[3]**	**Exp. val**	**Exp. val**	**Means**	**Exp. val**	**Exp. val**	**Exp. val**	**Means**
yhaK	2.26	0.01	15	13	12	13.33	39	33	19	30.33
c3917	1.62	0.01	50	53	68	57	78	92	107	92.33
deaD	1.57	3.93E-06	255	337	375	322.33	398	481	633	504
c1221	1.53	0.01	54	80	105	79.67	103	107	156	122
hrpA	1.4	1.72E-03	483	582	595	553.33	774	716	828	772.67
c1220	1.3	0.04	1,096.00	1,134.00	1,787.00	1,339.00	1,503.00	1,642.00	2,032.00	1,725.67
fimA	-1.3	0.04	940	954	1,333.00	1,075.67	782	727	967	825.33
focC	-1.37	0.04	262	331	250	281	204	180	234	206
sfaD	-1.42	0.01	233	314	244	263.67	186	173	201	186.67
focD	-1.45	7.59E-05	975	1,266.00	861	1,034.00	726	629	801	718.67
focH	-1.48	5.47E-04	320	418	309	349	217	207	286	236.67
focG	-1.53	1.01E-03	178	267	215	220	138	143	150	143.67
focA	-1.53	2.43E-07	3,394.00	4,230.00	3,335.00	3,653.00	2,300.00	2,210.00	2,693.00	2,401.00
sfaB	-1.98	1.27E-05	84	120	73	92.33	46	44	50	46.67
mtr	-2.53	4.57E-07	481	1,843.00	114	812.67	309	530	108	315.67
trpB	-2.6	1.28E-06	229	1,490.00	165	628	213	339	158	236.67
trpA	-2.61	2.42E-05	147	987	117	417	166	204	102	157.33
trpD	-5.96	4.76E-40	122	1,602.00	43	589	95	159	36	96.67
trpE	-8.2	1.39E-46	79	1,262.00	25	455.33	57	97	9	54.33
yhaJ	-651.51	4.57E-59	72	81	91	81.33	0	0	0	0

[1]Fold change derived from EdgeR normalised data

[2]FC-associated *p*-value with a false discovery rate (FDR) of 5% applied

[3]Expression values depicted as unique reads mapped per gene

Table S3. Summary of YhaJ^{FLAG} binding sites in EHEC identified by ChIP-seq

				Replicate 1					Replicate 2						
Gene[1]	Dist. Gene[2]	Dist. Centre[3]	Context[4]	Co-ordinates	Centre	Length[5]	PS score[6]	P-value	Co-ordinates	Centre	Length	PS score	*p*-value	MEME[7]	MEME *p*-value
yhhW	-196	-3	Intergenic	4366109..4366512	4366313	404	18	1.01E-72	4366106..4366487	4366297	382	26.76	4.91E-158	TCAATTTTTTTGAACA	2.10E-7
ybiJ	-412	-72	Intergenic	964223..964632	964433	410	13.19	4.71E-40	964237..964642	964452	406	20.82	1.44E-96	TCAACAGTACAATTGA	7.60E-2
yqjF	40	93	Intergenic	4057161..4057561	4057361	401	12.84	4.84E-38	4057166..4057560	4057370	395	17	3.84E-65	TCTTCAAAAAATTTGA	4.09E-8
yoaC	-305	-65	Intergenic	2570603..2571024	2570803	422	10.44	8.35E-26	2570607..2570995	2570798	389	21.14	1.68E-99	TTATAGATAAAATTGA	7.98E-5
z1211	-87	12	Intergenic	1131865..1132289	1132065	425	8.67	2.19E-18	1131874..1132300	1132065	427	16.28	6.37E-60	TTATCGATCGTTTTAT	3.63E-2
hslU	192	-16	Intragenic	4992143..4992553	4992354	411	6.7	1.07E-11	4992177..4992561	4992371	385	7.64	1.09E-14	TCGAACTGTTTGAACG	3.12E-3
yhaJ	-80	38	Intergenic	4060904..4061323	4061104	420	6.55	2.89E-11	4060852..4061276	4061088	425	8.15	1.76E-16	TCAAATTTCCTGAATG	1.89E-2
fimA	-132	-65	Intergenic	5427663..5428080	5427881	418	6.52	3.42E-11	5427641..5428071	5427881	431	8.08	3.23E-16	TCAAACTCGTTGACAA	9.13E-4
ybbF	-237	32	Intragenic	646099..646520	646321	422	6.38	8.66E-11	646098..646518	646289	421	8.93	2.07E-19	TCAACGTGGTTGATAA	2.04E-3
z1651	-64	26	Intergenic	1527497..1527897	1527697	401	6.37	9.67E-11	1527514..1527896	1527706	383	12.12	3.90E-34	TTATCGATCGTTTTAT	3.42E-2
hrpA	-44	-87	Intergenic	2089347..2089831	2089547	485	5.61	1.04E-08	2089379..2089775	2089570	397	10.17	1.29E-24	TCGAATTTATTGAACA	3.58E-7
pstB	110	50	Intragenic	4763714..4764143	4763944	430	4.37	6.15E-06	4763694..4764075	4763885	382	3.79	7.43E-05	ACAAAATGTTTGAACT	3.16E-5
tdcE	408	6	Intragenic	4068650..4069074	4068850	425	3.84	6.26E-05	4068645..4069060	4068836	416	4.24	1.13E-05	TGGACAGTGAATTTGA	2.10E-7
gadX	-100	29	Intergenic	4475067..4475476	4475267	410	3.76	8.66E-05	4475029..4475448	4475261	420	3.04	1.17E-03	TTATCAATCAATTTGA	1.30E-9
recJ	-249	28	Intragenic	3843604..3844006	3843807	403	3.51	2.23E-04	3843687..3844083	3843876	397	4.02	2.95E-05	ACAAAGCGTTTGATGA	2.00E-3
yedL	-150	-27	Intergenic	2707987..2708393	2708187	407	3.42	3.08E-04	2708031..2708419	2708222	389	5.88	2.09E-09	TTATCAAACAGGTTGA	3.58E-7
z1693	-168	85	Intergenic	1563238..1563688	1563438	451	3.1	9.54E-04	1563222..1563604	1563414	383	4.83	6.74E-07	TCAAAATTATTGAAAT	3.58E-7
tdk	424	-9	Intragenic	1830706..1831119	1830906	414	2.43	7.51E-03	1830732..1831115	1830923	384	5.18	1.13E-07	AATTCAACCAGTTTGT	1.69E-2
yejA	107	-4	Intergenic	3078467..3078899	3078667	433	2.38	8.57E-03	3078476..3078914	3078667	439	2.92	1.77E-03	TCAAAATGGTTGAAAT	6.54E-7
yecI	-267	19	Intergenic	2662830..2663242	2663043	413	2.36	9.26E-03	2662854..2663235	2663045	382	2.65	4.07E-03	TAATCAATGAATTTGA	1.35E-7
z2043	-324	65	Intergenic	1855675..1856181	1855875	507	2.33	9.78E-03	1855685..1856257	1855876	573	4.85	6.09E-07	ACAAACTTTTTGAAGA	3.58E-7
nleA	-461	-14	Intergenic	2283629..2284047	2283848	419	1.35	0.09	2283639..2284034	2283844	396	3.14	8.50E-04	TCTAACTCATTGATTA	1.90E-2
ftsA	511	6	Intragenic	108882..109292	109082	411	4.40E+00	5.47E-06	108910..109315	109101	406	6.76	7.03E-12	ACGATGTTTTTCGCCA	1.13E-6

[1]Gene with the 5’ end nearest to the associated peak
[2]Distance of the peak from the nearest gene 5’ end
[3]Distance of the predicted motif nearest the peak centre
[4]Genomic context of the peak
[5]Length of the peak window
[6]Peak shape score determined based on signal enrichment over negative control background
[7]Predicted LTTR motif (T-N^{11}-A; underlined) closest to peak centres and according to the YhaJ consensus determined in Palevsky *et al.* (2016) identified using MEME

Table S4. Summary of YhaJ^{FLAG} binding sites in UPEC identified by ChIP-seq

				Replicate 1					Replicate 2						
**Gene[1]**	**Dist. Gene[2]**	**Dist. Centre[3]**	**Context[4]**	**Co-ordinates**	**Centre**	**Length[5]**	**PS score[6]**	**P-value**	**Co-ordinates**	**Centre**	**Length**	**PS score**	***p*-value**	**MEME[7]**	**MEME *p*-value**
yhhW	-191	81	Intergenic	4018891..4019318	4019101	428	26.86	2.88E-159	4018891..4019310	4019117	420	11.29	7.34E-30	TCAATTTTTTTGAACA	1.77E-9
ybiJ	-442	241	Intergenic	860407..860877	860668	471	21.67	1.98E-104	860366..860881	860688	516	7.41	6.15E-14	TTATCAGTATGTTAGA	5.26E-6
c3206	285	5	Intragenic	3066009..3066460	3066219	452	16.41	8.39E-61	3066038..3066438	3066232	401	6.25	2.00E-10	TGAAGAAAAGCTTTGA	1.61E-6
yqjF	-44	9	Intergenic	3690291..3690719	3690501	429	13.5	7.66E-42	3690308..3690739	3690502	432	3.78	7.80E-05	TTTTCAAAAAATTTGA	9.14E-10
yhaJ	-22	-3	Intergenic	3693391..3693819	3693610	429	9.12	3.69E-20	3693343..3693862	3693669	520	3.24	5.89E-04	TCAAATTTCCTGAATG	1.34E-6
yoaC	283	-66	Intergenic	2045235..2045664	2045445	430	7.22	2.52E-13	2045249..2045656	2045463	408	2.29	0.01	TTATAGATAAAATTGA	1.99E-6
purH	542	-12	Intragenic	4737946..4738388	4738156	443	8.61	3.73E-18	4737966..4738361	4738168	396	2.27	0.01	TCAAAGCCTTTGAACA	1.59E-7

[1]Gene with the 5' end nearest to the associated peak

[2]Distance of the peak from the nearest gene 5' end

[3]Distance of the predicted motif nearest the peak centre

[4]Genomic context of the peak

[5]Length of the peak window

[6]Peak shape score determined based on signal enrichment to noise ratio

[7]Predicted LTTR motif (T-N^{11}-A; underlined) closest to peak centres and according to the YhaJ consensus determined in Palevsky *et al.* (2016) identified using MEME

Table S5. Bacterial strains used in this study.

**Name**	**Description**	**Source**
EHEC	Enterohaemorrhagic *E. coli* strain TUV93-0 (Stx -ve)	Roe lab inventory
UPEC	Uropathogenic *E. coli* strain CFT073	Roe lab inventory
K-12	*E. coli* strain MG1655	Roe lab inventory
$\Delta \mathit{yhaJ}^{\mathrm{EHEC}}$	EHEC *yhaJ* deletion mutant	Connolly *et al.,* 2016
$\Delta \mathit{yhaJ}^{\mathrm{UPEC}}$	UPEC *yhaJ* deletion mutant	This study
$\Delta \mathit{yhaJ}^{\mathrm{K\text{-}12}}$	K-12 *yhaJ* deletion mutant	This study
EHEC $\mathrm{YhaJ}^{\mathrm{FLAG}}$	EHEC YhaJ-FLAG native fusion	This study
UPEC $\mathrm{YhaJ}^{\mathrm{FLAG}}$	UPEC YhaJ-FLAG native fusion	This study
BL21-DE3	Commercial protease deficient *E. coli* for T7 expression	Invitrogen

Table S6. Plasmids used in this study.

**Name**	**Description**	**Reference**
pKD46	Encodes λ recombinase system; propagates at 30 ℃; ampicillin resistant.	(4)
pCP20	Encodes FLP recombinase; propagates at 30 ℃; ampicillin resistant.	(4)
pKD3	Carries FRT-flanked chloramphenicol cassette with downstream RBS; Pir dependent*;* ampicillin resistant.	(4)
pKD4	Carries FRT-flanked kanamycin cassette with downstream RBS; Pir dependent*;* ampicillin resistant.	(4)
pDOC-F	Carries FRT-flanked kanamycin cassette of pKD4 preceded by 3X FLAG tag; I-*Sce*I cleavable; ampicillin resistant.	(5)
pBAD18	Encodes inducible arabinose promoter downstream of the MCS; ampicillin resistant.	(6)
p*yhaJ*^{EHEC}	EHEC *yhaJ* coding sequence cloned into pBAD18 with *Nhe*I/*Hind*III.	This study
p*yhaJ*^{UPEC}	UPEC *yhaJ* coding sequence cloned into pBAD18 with *Nhe*I/*Hind*III.	This study
pET28-*yhaJ*	*yhaJ* coding sequence fused in frame to a 6X Histidine tag expressed in pET28.	(13)
pWSK-*yhaJ*	EHEC *yhaJ* coding sequence cloned into pWSK29	(13)
p*nleA*::GFP	EHEC *nleA* promoter region-GFP fusion reporter in pAJR70; chloramphenicol resistant.	(16)
p*LEE1*::GFP	EHEC *LEE1* promoter region-GFP fusion reporter in pAJR70; chloramphenicol resistant.	(15)
p*yhaJ*::GFP	K-12 *yhaJ* promoter region-GFP fusion reporter in pUA66; kanamycin resistant.	(17)
pSR	2.6 kb; *Eco*RI-*Hind*III restriction cloning site, upstream of a λoop; ampicillin resistant.	(7)
pSR-*yhhW*	UPEC *yhhW* ChIP-seq peak region cloned into pSR.	This study
pSR-*fimSOFF*	UPEC *fimS* phase OFF ChIP-seq peak region cloned into pSR.	This study

Table S7. Oligonucleotide primers used in this study.

Name	Description	Sequence
UPEC_yhaJ_KO_F	pKD3/4 template forward primer containing CFT073 *yhaJ* upstream flanking sequence	TTAGCCAGATATTCTGCCCGGTATGTTCAAATTTCCTGAATGAGAACGAAGTGTAGGCTGGAGCTGCTTC
UPEC_yhaJ_KO_R	pKD3/4 template reverse primer containing CFT073 *yhaJ* downstream flanking sequence	AACTGGAACAAGACTAATAATTAAGCACCTTGTAAAAGGTGCTTAATCGACATATGAATATCCTCCTTAG
UPEC_yhaJ_check_F	CFT073 *yhaJ* insert check forward primer	CTTCCTTTTATGGTAAGGGG
UPEC_yhaJ_check_R	CFT073 *yhaJ* insert check reverse primer	TGGCTTATTATCATCGTCTTT
K12_yhaJ_KO_F	pKD3/4 template forward primer containing MG1655 *yhaJ* upstream flanking sequence	TTAGCCAGATATTCTGCCCGGTATGTTCAAATTTCCTGAATGAGAACGAAGTGTAGGCTGGAGCTGCTTC
K12_yhaJ_KO_R	pKD3/4 template reverse primer containing MG1655 *yhaJ* downstream flanking sequence	TCTTAGAATTGGGGCGATATTTCGCCCCTTTTTATTAACAATAATAAATACATATGAATATCCTCCTTAG
K12_yhaJ_check_F	MG1655 *yhaJ* insert check forward primer	TTAGCCAGATATTCTGCCCG
K12_yhaJ_check_R	MG1655 *yhaJ* insert check reverse primer	GCGATATTTCGCCCCTTT
EHEC_yhaJFLAG_F	pDOC-F template forward primer containing TUV93-0 *yhaJ* upstream stop codon flanking sequence	AAGCGAAATCCTGGTGTCTGCGGGAAATTCCCAAACTTTTTAACGGAAAAGACTACAAAGACCATGACGG
EHEC_yhaJFLAG_R	pDOC-F template reverse primer containing TUV93-0 *yhaJ* downstream stop codon flanking sequence	TCTTAGAATTGGGGCGATATTTCGCCCCTTTTTATTAACAATAATAAATAAATATCCTCCTTAGTTCC
UPEC_yhaJFLAG_F	pDOC-F template forward primer containing CFT073 *yhaJ* upstream stop codon flanking sequence	AGGCGAAATCCTGGTGTCTGCGGGAAATTCCCAAACTTTTTAGCGGAAAAGACTACAAAGACCATGACGG
UPEC_yhaJFLAG_R	pDOC-F template reverse primer containing CFT073 *yhaJ* downstream stop codon flanking sequence	AACTGGAACAAGACTAATAATTAAGCACCTTGTAAAAGGTGCTTAATCGAAATATCCTCCTTAGTTCC
EHEC_UPEC_yhaJFLAG_check_F	TUV93-0/CFT073 *yhaJ*-FLAG insert check forward primer	GACCAGCGAGATCGATATTA
EHEC_yhaJFLAG_check_R	TUV93-0 *yhaJ*-FLAG insert check reverse primer	AGGTACTTCTGGCAGCAATC
UPEC_yhaJFLAG_check_R	CFT073 *yhaJ*-FLAG insert check reverse primer	GTCTTTAACTGCCAGGCGGG
pBAD_yhaJ_NheI_F	Forward primer containing NheI for *yhaJ* ligation into pBAD18	CCCGCTAGCCAAATTTCCTGAATGAGAAC
pBAD_yhaJ_EHEC_HindIII_R	Reverse primer containing HindIII for *yhaJ* ligation into pBAD18	CCCAAGCTTTATTTTCCGTTAAAAAG

pBAD_yhaJ_UPEC_HindIII_R	Reverse primer containing HindIII for *yhaJ* ligation into pBAD18	CCCAAGCTTTTATTTTCCGCTAAAAAG
pBAD18_check_F	pBAD18 insert check forward primer	TGCCATAGCATTTTTATCCA
pBAD18_check_R	pBAD18 insert check reverse primer	CGCTTCTGCGTTCTGATTT
fimA_qPCR_F	*fimA* forward primer for qRT-PCR	ATCGTTGTTCTGTCGGCTCT
fimA_qPCR_R	*fimA* reverse primer for qRT-PCR	TTAAAGTGAACGGTCCCACC
fimE_qPCR_F	*fimE* forward primer for qRT-PCR	AGAAGTTCAGGCCATGATGC
fimE_qPCR_R	*fimE* reverse primer for qRT-PCR	ATGCCGATATGCCAACAGA
gadB_qPCR_F	*gadB* forward primer for qRT-PCR	GACGATGTCGCATTTCAGAT
gadB_qPCR_R	*gadB* reverse primer for qRT-PCR	AGGTCTGGCAGAAAGTGGC
LEE1_qPCR_F	*LEE1* forward primer for qRT-PCR	GACTGCGAGAGCAGGAAGTT
LEE1_qPCR_R	*LEE1* reverse primer for qRT-PCR	ATCCCAGCTCTTGTAAGGTT
nleA_qPCR_F	*nleA* forward primer for qRT-PCR	TAGGATGCCAAGCTGGATTT
nleA_qPCR_R	*nleA* reverse primer for qRT-PCR	AGCTGTTGTTTCACCGCATT
gadX_qPCR_F	*gadX* forward primer for qRT-PCR	ATTTTAATGGCGGTGACCTG
gadX_qPCR_R	*gadX* reverse primer for qRT-PCR	CGGGAGAAATAATGAAAGCG
EHEC_UPEC_T1P_F	TUV93-0/CFT073 *fimS* phase orientation forward primer	GAGAAGAAGTTTGATTTAACTTATTG
EHEC_K12_T1P_R	TUV93-0/MG1655 *fimS* phase orientation reverse primer	AGAGCCGCTGAACTGAGG
UPEC_T1P_R	CFT073 *fimS* phase orientation reverse primer	AGAGCCGCTGTGGAACTGAGG
K12_T1P_F	MG1655 *fimS* phase orientation forward primer	GAGAAGAGGTTTGATTTAACTTATTG
yhaK_ChIP_F	*yhaK* ChIP-PCR forward pimer	GGCCATTTCGTTCTCATT
yhaK_ChIP_R	*yhaK* ChIP-PCR reverse pimer	CCTTTTATGGTAAGGGGC
yhhW_ChIP_F	*yhhW* ChIP-PCR forward pimer	ACTTGAACGTGGATTCGAGC
yhhW_ChIP_R	*yhhW* ChIP-PCR reverse pimer	GGGTGAAAATTGACCCCTCT
yqjF_ChIP_F	*yqjF* ChIP-PCR forward pimer	CGGCCTTACCCATCAAAT
yqjF_ChIP_R	*yqjF* ChIP-PCR reverse pimer	AGAATCATAAACGTGGTGAA
araD_ChIP_F	*araD* control ChIP-PCR forward pimer	CTGCCAAAACACAACCTG
araD_ChIP_R	*araD* control ChIP-PCR reverse pimer	TTTGATCACAAAGACGCC
nleA_EMSA_F	*nleA* ChIP region forward primer	AATCTGATTCAATGGCGGC
nleA_EMSA_R	*nleA* ChIP region reverse primer	GACATATAATGCTTGGCAGC
gadX_EMSA_F	*gadX* ChIP region forward primer	GAATGTTATGGAGCATAGCG

gadX_EMSA_R	*gadX* ChIP region reverse primer	ATCCGCAAAAACCAGATCAC
c3205_EMSA_F	c3205 ChIP region forward primer	GGATGTTACGTGAGATTGAAGC
c3205_EMSA_R	c3205 ChIP region reverse primer	TCGACCTGGGAAACAACTTC
Z1211_EMSA_F	Z1211 ChIP region forward primer	ACAAAAGTGGATACCGGCAG
Z1211_EMSA_R	Z1211 ChIP region reverse primer	ACAGTTCTGCGTGCCGTCCGTGACA
Z2043_EMSA_F	Z2043 ChIP region forward primer	ATTATTCGCACTGAGCCAGG
Z2043_EMSA_R	Z2043 ChIP region reverse primer	AGCCTAAGAGTTATGGTCAAGC
fimS_ON_EMSA_F	*fimS* phase ON region forward primer	GCGGAGGTGATGTGAAATTA
fimS_OFF_EMSA_F	*fimS* phase OFF region forward primer	CTCCAAAAACCACCTCAT
fimS_ON_450_EMSA_F	*fimS* phase ON 450 bp fragment forward primer	CTCATAGAGGAAAGCATCGC
fimS_ON_250_EMSA_F	*fimS* phase ON 250 bp fragment forward primer	CATGAGGTGGTTTTTGGAG
fimS_OFF_118_285_EMSA_F	*fimS* phase OFF 118/285 bp fragment forward primer	CTCCAAAAACCACCTCATG
fimS_external_EMSA_R	*fimS* including fimA intergenic region forward primer	AGAGCCGACAGAACAACGAT
fimS_internal_OFF_R	*fimS* excluding fimA intergenic region forward primer	CTCATAGAGGAAAGCATCGC
yhhW_pSR_F	*yhhW* ChIP region forward primer with EcoRI restriction site for ligation into pSR	CCGAATTCGGATGATTCGGTCGGTGT
yhhW_pSR_R	*yhhW* ChIP region reverse primer with HindIII restriction site for ligation into pSR	CCAAGCTTCCTGGGATGATAGAGGGTGA
fimS_OFF_pSR_F	*fimS* ChIP region forward primer with EcoRI restriction site for ligation into pSR	CCGAATTCCTCCAAAAACCACCTCAT
fimS_OFF_pSR_R	*fimS* ChIP region reverse primer with HindIII restriction site for ligation into pSR	CCAAGCTTAGAGCCGACAGAACAACGAT

Table S8. RNA-seq mapping statistics from this study.

Sample	Total reads	Unique reads	% unique	Non-specific	% non-specific	Uncounted	% uncounted
UPEC WT 1	10356366	2225716	21.49	8104772	78.26	25878	0.25
UPEC WT 2	10868437	2473403	22.76	8372211	77.03	22823	0.21
UPEC WT 3	11356635	2569315	22.62	8754420	77.09	32900	0.29
Δ*yhaJ*^{UPEC} 1	10412044	2367508	22.74	8021692	77.04	22844	0.22
Δ*yhaJ*^{UPEC} 2	11636545	2470528	21.23	9137866	78.53	28151	0.24
Δ*yhaJ*^{UPEC} 3	11246264	2571267	22.86	8647562	76.89	27435	0.24
EHEC WT1	13994113	1286147	9.19	12516053	89.44	191913	1.37
EHEC WT2	12225585	1078631	8.82	10966097	89.7	180857	1.48
EHEC WT3	14237184	1182144	8.3	12833295	90.14	221745	1.56
Δ*yhaJ*^{EHEC} 1	14164082	1177677	8.31	12803155	90.39	183250	1.29
Δ*yhaJ*^{EHEC} 2	13083953	1057642	8.08	11802924	90.21	223387	1.71
Δ*yhaJ*^{EHEC} 3	13524032	1149347	8.5	12179818	90.06	194867	1.44

Supplementary references

1. Perna NT, et al. (2001) Genome sequence of enterohaemorrhagic Escherichia coli O157:H7. *Nature* 409:529–533.
2. Welch RA, et al. (2002) Extensive mosaic structure revealed by the complete genome sequence of uropathogenic Escherichia coli. *Proc Natl Acad Sci U S A* 99(26):17020–4.
3. Blattner FR, et al. (1997) The complete genome sequence of Escherichia coli K-12. *Science* 277(5331):1453–1462.
4. Datsenko KA, Wanner BL (2000) One-step inactivation of chromosomal genes in Escherichia coli K-12 using PCR products. *Proc Natl Acad Sci U S A* 97(12):6640–5.
5. Lee DJ, et al. (2009) Gene doctoring: A method for recombineering in laboratory and pathogenic Escherichia coli strains. *BMC Microbiol* 9(52). doi:10.1186/1471-2180-9-252.
6. Guzman LM, Belin D, Carson MJ, Beckwith J (1995) Tight regulation, modulation, and high-level expression by vectors containing the arabinose P(BAD) promoter. *J Bacteriol* 177(14):4121–4130.
7. Kolb A, Kotlarz D, Kusano S, Ishihama A (1995) Selectivity of the Escherichia coli RNA polymerase Eσ38for overlapping promoters and ability to support CRP activation. *Nucleic Acids Res* 23(5):819–826.
8. Bonocora RP, Wade JT (2015) ChIP-Seq for Genome-Scale Analysis of Bacterial DNA-Binding Proteins. *Methods in Molecular Biology* (Humana Press, New York, NY), pp 327–340.
9. Robinson MD, McCarthy DJ, Smyth GK (2010) edgeR: a Bioconductor package for differential expression analysis of digital gene expression data. *Bioinformatics* 26(1):139–40.

10. Strino F, Lappe M (2016) Identifying peaks in *-seq data using shape information. *BMC Bioinformatics* 17(Suppl 5):206.

11. Bailey TL, et al. (2009) MEME Suite: Tools for motif discovery and searching. *Nucleic Acids Res* 37(Suppl 2):202–208.

12. Livak KJ, Schmittgen TD (2001) Analysis of relative gene expression data using real-time quantitative PCR and the 2-ΔΔCT method. *Methods* 25(4):402–8.

13. Connolly JPR, et al. (2016) A Highly Conserved Bacterial D-Serine Uptake System Links Host Metabolism and Virulence. *PLOS Pathog* 12(1):e1005359.

14. Browning DF, Cole JA, Busby SJW (2008) Regulation by nucleoid-associated proteins at the Escherichia coli nir operon promoter. *J Bacteriol* 190(21):7258–7267.

15. Roe AJ, et al. (2003) Heterogeneous surface expression of EspA translocon filaments by Escherichia coli O157:H7 is controlled at the posttranscriptional level. *Infect Immun* 71(10):5900–5909.

16. Roe AJ, et al. (2007) Analysis of the expression, regulation and export of NleA-E in Escherichia coli O157:H7. *Microbiology* 153(Pt 5):1350–60.

17. Zaslaver A, et al. (2006) A comprehensive library of fluorescent transcriptional reporters for Escherichia coli. *Nat Methods* 3(8):623–8.

18. Chen SL, et al. (2009) Positive selection identifies an in vivo role for FimH during urinary tract infection in addition to mannose binding. *Proc Natl Acad Sci* 106(52):22439–22444.

19. Chaudhuri RR, Pallen MJ (2006) xBASE, a collection of online databases for bacterial comparative genomics. *Nucleic Acids Res*. doi:10.1093/ar/gkj140.

20. Connolly JPR, et al. (2015) The host metabolite D-serine contributes to bacterial niche specificity through gene selection. *ISME J* 9:1039–1051.

21. Sullivan MJ, Petty NK, Beatson SA (2011) Easyfig: A genome comparison visualizer. *Bioinformatics* 27:1009–1010.

22. Fruzangohar M, et al. (2013) Comparative GO: A Web Application for Comparative Gene Ontology and Gene Ontology-Based Gene Selection in Bacteria. *PLoS One* 8(3):e58759.

23. Palevsky N, Shemer B, Connolly JPR, Belkin S (2016) The highly conserved Escherichia coli transcription factor yhaJ regulates aromatic compound degradation. *Front Microbiol*. doi:10.3389/fmicb.2016.01490.

24. Zhang H, Susanto TT, Wan Y, Chen SL (2016) Comprehensive mutagenesis of the fimS promoter regulatory switch reveals novel regulation of type 1 pili in uropathogenic Escherichia coli. *Proc Natl Acad Sci* 113(15):4182–4187.
